# Recent metaheuristic algorithms for solving some civil engineering optimization problems

**DOI:** 10.1038/s41598-025-90000-8

**Published:** 2025-03-07

**Authors:** Essam H. Houssein, Mohamed Hossam Abdel Gafar, Naglaa Fawzy, Ahmed Y. Sayed

**Affiliations:** 1 Faculty of Computers and Information, Minia University, Minia, Egypt; 2 Minia National University, Minia, Egypt; 3 Physics and Engineering Mathematics Department, Faculty of Engineering at Mataria, Helwan University, Cairo, Egypt

**Keywords:** Metaheuristic optimization algorithms, Blade eagle search, Welded beam design, Civil engineering, Civil engineering, Computational science

## Abstract

In this study, a novel hybrid metaheuristic algorithm, termed (BES–GO), is proposed for solving benchmark structural design optimization problems, including welded beam design, three-bar truss system optimization, minimizing vertical deflection in an I-beam, optimizing the cost of tubular columns, and minimizing the weight of cantilever beams. The performance of the proposed BES–GO algorithm was compared with ten state-of-the-art metaheuristic algorithms: Bald Eagle Search (BES), Growth Optimizer (GO), Ant Lion Optimizer, Tuna Swarm Optimization, Tunicate Swarm Algorithm, Harris Hawk Optimization, Artificial Gorilla Troops Optimizer, Dingo Optimizer, Particle Swarm Optimization, and Grey Wolf Optimizer. The hybrid algorithm leverages the strengths of both BES and GO techniques to enhance search capabilities and convergence rates. The evaluation, based on the CEC’20 test suite and the selected structural design problems, shows that BES–GO consistently outperformed the other algorithms in terms of convergence speed and achieving optimal solutions, making it a robust and effective tool for structural Optimization.

## Introduction

Many real-world problems can be modelled as optimization problems. Optimization could be described as the process of selecting of the best element(s) from among a set of available alternatives to get the best possible results when solving a particular problem. Optimization algorithms attempt to reach the optimal objective values (i.e., minimum, or maximum) and satisfy the related constraints. Optimization methods can be classified into two main groups: deterministic^1^ and non-deterministic methods. Deterministic methods based on mathematical modelling and programming techniques initiate the search process from an initial design point, typically by computing the gradient of the objective function. These methods aim to explore the search space towards the optimal point. Although they distinguish with a rapid convergence rate and high accuracy, their effectiveness relies heavily on the starting point. But they have some disadvantages which is the objective function must be continuous or partially continuous along with its gradients which cannot be possible for many engineering problems. The non-deterministic approaches are considered as an alternative optimization approach which overcomes the disadvantages of deterministic methods. Because they are based on probabilistic rules of transition rather than deterministic ones^2^.The non-deterministic approach can be categorized into heuristics and metaheuristics. The significant difference between both is that heuristics are more problem-dependent than metaheuristics.

In other words, heuristics can be efficiently applied to a specific problem meanwhile become insufficient to other problems. On the other hand, a metaheuristic is a generic algorithm framework or a black box optimizer that can be applied to all optimization problems^3^. The term “metaheuristics” refers to a “higher level of heuristics” and is a combination of the words “meta” and “heuristic,” where “meta” means beyond or higher level and “heuristic” refers to finding or discovering a goal by trial and error.

Metaheuristic algorithms are optimization algorithms that are used to find the optimal solution for complex problems that cannot be solved using traditional methods^4^ like travelling salesman problem^5^, the knapsack optimization problem^6^ and Bin Packing Problem^7^ Which are non-deterministic polynomial-time-hard combinatorial problems. Metaheuristics optimization can be classified into four main categories: Evolution-based, physics-based, swarm-based, and human-based, each of which simulates specific behaviours for proposing a novel algorithm with distinct characteristics (Exploration and exploitation). Evolution-based algorithms are based on the genetic characteristics and evolutionary methods of nature, and representative algorithms include ES, evolutionary programming (EP), genetic algorithm (GA), and differential evolution (DE). Swarm-based algorithms are based on the behaviour of organisms such as birds or ants in clusters, and representative algorithms include ant colony optimization (ACO), particle swarm optimization (PSO), cuckoo search, and crow search (CS). Physical-based algorithms are based on physical phenomena, and representative algorithms include simulated annealing (SA), harmony search (HS), gravitational search (GS), black hole (BH), and sine cosine (SC). Finally, the human behaviour-based algorithms are based on human intelligent behaviour, and representative algorithms include human-inspired (HI), social-emotional optimization (SEO), brainstorm optimization (BSO), teaching learning-based optimization (TLBO), and social-based (SB)^8^. They employ several randomly generated agents and gradually improve them until the convergence/termination condition is met. Metaheuristic approaches, as main the techniques belonging to this group, are inspired by natural phenomena. The basic clue behind these methods is to model natural concepts, like the survival behaviour of the animal colonies, physical rules etc. The population-based optimization algorithms are a powerful tool to reach global optimum solutions to real-world problems for 30 years have been commonly used to design optimal components^9^. Metaheuristic Optimization algorithms can solve many civil engineering optimization problems. For example, Optimum detailed design of reinforced concrete frames^10^.

In structural steel design optimization^11^, or implement the performance-based optimum seismic design of steel dual-braced frames for various performance levels by bat algorithm (BA), It also used in the asphalt pavement management system in transport infrastructure planning or geotechnical engineering optimization problems^12^ and many civil engineering optimization problems. Metaheuristic optimization algorithms solve efficiently many structural engineering problems which is one of civil engineering’s crucial branches. One of the primary objectives for structural engineers is a cost-effective design. Incorporating optimality criteria into the design procedure introduces additional complexities that result in problems that are nonlinear, nonconvex, and have a discontinuous solution space.^13^.

With the increase in the number of recent metaheuristics, the necessity of determining the best algorithm which solves optimization problems is questioned, many papers made comparisons to find the best algorithm according to convergence rate and minimum fitness for example a comparative investigation of eight recent population-based optimisation algorithms for mechanical and structural design problems^9^, comparison of recent meta-heuristic optimization algorithms using different benchmark functions^14^ and comparative assessment of five metaheuristic methods on distinct problems^2^ each paper study some of metaheuristic optimizations algorithms and compare between their results as (best, mean, worst, average, standard deviation and function evaluation).

After this comparison, the better algorithms can be used in many applications like the Effect of bar diameter on bond performance of helically ribbed GFRP bar to UHPC^15^, Mechanical properties and microstructure of waterborne polyurethane-modified cement composites as concrete repair mortar^16^, Reaction molecular dynamics study of calcium alumino-silicate hydrate gel in the hydration deposition process at the calcium silicate hydrate interface: The influence of Al/Si^17^, Durability enhancement of cement-based repair mortars through waterborne polyurethane modification: Experimental characterization and molecular dynamics simulations^18^ and Unveiling the dissolution mechanism of calcium ions from CSH substrates in Na2SO4 solution: Effects of Ca/Si ratio^19^

This paper’s main contributions come from the creation of a novel hybrid algorithm from Blade Eagle Search (BES)^20^, and Growth Optimizer (GO)^21^. Then it is compared to ten well-metaheuristic algorithms, selected for their noteworthy progress and success between 2009 and 2023. The comparison is conducted using ten benchmark functions CEC2020 and five challenging real-world structural design problems. These problems are the welded beam design, weight optimization of a cantilever beam, I-beam vertical deflection, tubular column design optimization, and the three-bar truss system optimization.

These issues present diverse levels of constraint difficulty and intricacies within their respective search spaces. The hybrid BES–GO algorithm is evaluated against the other ten algorithms to determine the best optimal value, minimum standard deviation, best average value, and highest convergence rate over 30 independent runs. These algorithms are Blade Eagle Search (BES), Growth Optimizer (GO), Ant Lion Optimiser (ALO)^22^, Tuna Swarm Optimization (TSO)^23^, Tunicate Swarm Algorithm (TSA)^24^, Harris Hawks Optimization (HHO)^25^, Artificial Gorilla Troops Optimizer (GTO)^26^, Dingo Optimizer (DOA)^27^, Particle swarm optimizations (PSO)^28^, Grey Wolf Optimizer (GWO)^29^.

The rest of this paper is organized as follows. Section “Related works” offers an extensive review of metaheuristic algorithms published in the last five years, along with their outcomes in solving five optimization problems. Section “Metaheuristics algorithms” provides a comprehensive review of the selected metaheuristic algorithms and their respective methodologies including BES and GO as the basis for the hybrid metaheuristic algorithm BES–GO. Section “BES–GO algorithm” explains the detailed structure of BES–GO. Section “Engineering structural design problems” provides a comprehensive overview of the selected optimization problems including objective functions constraints and benchmark functions CEC2020. Section “Experimental results analysis” provides the results of the implementation of these algorithms. Section “Nonparametric statistical analysis” provides a nonparametric statistical analysis for the BES–GO algorithm. Section “Conclusion and future work” provides the conclusion and future work.

## Related works

Numerous population-based optimization methods are employed to tackle benchmark optimization problems such as the welded beam design problem, 3 Bar design problem, cost optimization of tubular columns, and vertical deflection minimization problem of an I-beam. This section will highlight significant endeavours in this domain. Various algorithms, including the Colliding Bodies Optimization Algorithm^30^, Social Spider Optimization (SSO-C)^31^, and the New Movement Strategy of Cuckoo Search (NMS-CS)^32^ For many other algorithms Table 1 presents recent metaheuristic algorithms along with their optimal solutions and corresponding fitness values when applied to solving the welded beam design problem.

**Table 1:** Summary of related works and algorithms to solve welded beam design problem and their results.

References	Algorithm	Best fitness	Solution			
			h	l	t	b
^30^	CBO	1.72466	0.20572	3.47041	9.03728	0.20574
^31^	SSO-C	1.72485	0.20572	3.47048	9.03662	0.20573
^32^	NMS-CS	1.72620	0.20549	3.47833	9.03945	0.20575
^33^	AZOA	1.72000	0.46900	1.94000	5.72000	0.51400
^34^	ABC	1.72485	0.20573	3.47049	9.03662	0.20573
^35^	Bat algorithm	1.78519	0.20267	3.52714	9.00752	0.21053
^36^	WCMFO	1.72358	0.20671	3.44955	9.03679	0.20573
^37^	ASOINU	2.08685	0.17533	7.95699	9.84772	0.17324
^38^	HSOGA	1.72734	0.20392	3.50976	9.03669	0.20573
^39^	LEA	1.72500	–	–	–	–
^40^	FDA	1.69550	0.20550	3.25780	9.03660	0.20570
^41^	MSHO	1.72485	0.20573	3.470 471	9.03663	0.20573
^42^	ACSA	1.72490	0.20570	3.47050	9.03660	0.20570
^43^	Halton-PSO	1.57140	0.14450	2.83650	9.03610	1.57140
^44^	MSCA	2.38329	0.24425	6.20637	8.31217	0.24432
^45^	CEBA	1.69778	0.20473	3.27347	9.03901	0.20584

The three-bar truss design problem is renowned as a constrained design problem that has garnered significant attention in academic literature. Numerous papers have endeavoured to tackle this challenge, employing methodologies such as the American Zebra Optimization Algorithm^37^, along with several other algorithms documented in the subsequent Table 2.

**Table 2:** Summary of related works and algorithms solve three‑bar truss design problem and their results.

References	Algorithm	Best fitness	Solution	
			A1	A2
^35^	BAT	263.98439	0.786238	0.415372
^46^	AO	263.8684	0.7926	0.3966
^47^	AOA	263.9154	0.79369	0.39426
^40^	FDA	263.895843	0.788662	0.408286
^48^	SKF	263.8958	0.7887	0.4083
^41^	mSHO	263.8915	0.788 649	0.408 235
^49^	INFO	263.8958434	0.788672734	0.408255081
^50^	RUN	263.8958	0.788679110	0.408237045
^42^	ACSA	263.8958	0.78867	0.40825
^51^	SNS	263.8958434	0.78868473	0.4082211
^43^	Halton-PSO	263.8958	0.7887	0.4083
^44^	MSCA	263.89585052	0.788690415	0.408205144
^52^	EOBL-GOA	263.895	0.788665414	0.40827578
	OBLGOA	263.895844	0.78866365	0.408280786
^53^	EO	263.8954081	0.788674018	0.408242743
	COOT	263.8954081	0.788672536	0.408246935
	POA	263.8954081	0.613176	0.047329
	CA	263.8954081	0.303088	0.936761

Additionally, there exist numerous algorithms that address the cantilever beam optimization problem, as detailed in Table 3.

**Table 3:** Summary of related works and algorithms to solve cantilever beam optimization and their results.

References	Algorithm	Best Fitness	Solution				
			x (1)	x (2)	X (3)	X (4)	X (5)
^52^	EOBL-GOA	1.3399	6.01513	5.30930	5.000	3.501426	2.15278
^54^	GWOWOA	1.3399	6.0089	5.3081	4.4900	3.4950	2.1512
^55^	IRSHHO	1.339	6.073	5.110	4.658	3.602	2.103
^41^	mSHO	1.339 956	6.015 906	5.308 734	4.495 939	3.500 899	2.152 182
^46^	AO	1.3390	5.8881	5.5451	4.3798	3.5973	2.1026
^50^	RUN	1.3399563	6.0049	5.3190	4.4868	3.5033	2.1595
^56^	IBMs	1.33991812032	6.0157385	5.3090857	4.4927465	3.5019894	2.1534867
^57^	SaISOS	1.33515	5.929337	5.314156	4.449033	3.473583	2.1616463
^42^	ACSA	1.3399	6.0160	5.3092	4.4943	3.5014	2.1527
^51^	SNS	1.33995	6.01545	5.31066	4.48800	3.50528	2.15428
^53^	POA	1.339958155	45.79272	74.26591	74.93475	69.57448	76.50655
	CA	1.339957392	78.03137	23.95073	64.59176	63.55044	5.513643

The vertical deflection on an I-beam problem presents a considerable challenge, motivating numerous researchers to utilize diverse algorithms to showcase the effectiveness of their approaches. These endeavours are summarized in Table 4, highlighting notable discrepancies in the optimal results achieved by the different methodologies employed.

**Table 4:** Summary of related works and algorithms to solve vertical deflection on an I-beam problem and their results.

References	Algorithm	Best Fitness	Solution			
			h	b	t_w_	t_f_
^49^	INFO	0.0130741	80	50	0.90	2.32
^56^	ImBSA	0.007100487920316	80	50	1.7646138	5.0000000
^57^	SaISOS	0.0524	15.860770	49.9995421	2.7882569	4.9995440
^42^	ACSA	0.01229	80.0000	50.0000	1.5000	5.0000
^51^	SNS	0.0130741	80	50	0.9	2.3217
^43^	Halton-PSO	0.0066	80	50	1.7647	5
^44^	MSCA	0.01307412	80	50	0.900000012	2.32179198
^45^	CEBA	0.006625958	80	50	1.764706	5

Few researchers have attempted to solve the cost optimization of the tubular column problem, and their endeavours are documented in Table 5.

**Table 5:** Summary of related works and algorithms solve cost optimization of the tubular column.

References	Algorithm	Best Fitness	Solution	
			D	t
^38^	HSOGA	26.53132788013	5.451156	0.291965
^42^	ACSA	26.4995	5.45116	0.29197
^51^	SNS	26.4994969	5.45115623	0.29196547
^53^	POA	26.48636047	7.706642	0.767859
	CA	26.48636047	4.573024	0.633764
	MFO	26.48636047	5.452181287	0.291626342
	COOT	26.48636047	5.452181285	0.291626342

## Metaheuristics algorithms

In this section, a brief description of the algorithms and the parameter settings utilized in this study are provided. For more details and the literature, readers can go through the cited articles.

### Bald eagle search algorithm (BES)

The Bald Eagle Search (BES) algorithm, established in 2020^20^, mimics the intelligent hunting prowess of Bald Eagles to tackle challenging optimization problems. Inspired by their broad-scale scouting, prey localization, and precise dives, BES divides its search into three stages: exploring the search space, zeroing in on promising areas, and finally swooping down for the optimal solution. First stage is select stage, where bald eagles identify and select the best area (in terms of amount of food) within the selected search space where they can hunt for prey. Equation (1) presents this behaviour mathematically.

1 $${P}_{new,i}={P}_{best}+\alpha *r\left({P}_{mean}-{P}_{i}\right)$$

where ${P}_{new,i}$ is the updated position, ${P}_{best}$ denotes the search space that is currently selected by bald eagles based on the best position identified during their previous search, $\alpha$ is the parameter for controlling the changes in position that takes a value between 1.5 and 2 and $r$ is a random number that takes a value between 0 and 1, ${P}_{mean}$ indicates that these eagles have used up all information from the previous points. ${P}_{i}$ is the current position. The second stage is searching for stage where bald eagles search for prey within the selected search space and move in different directions within a spiral space to accelerate their search. The best position for the swoop is mathematically expressed in the following equation:

2 $${P}_{new,i}={P}_{be{st}^{+}}+y\left(i\right)*\left({P}_{mean}-{P}_{i+1}\right)+x\left(i\right)*({{P}_{i}- P}_{mean})$$

3 $$x\left(\text{i}\right)=\frac{{x}_{r}\left(\text{i}\right)}{\text{max}\left|xr\right|}$$

4 $$y\left(i\right)=\frac{{y}_{r}\left(i\right)}{\text{max}\left|yr\right|}$$

5 $$xr\left(i\right)=r\left(i\right)*\text{sin}\left(\theta \left(i\right)\right)$$

6 $$yr\left(\text{i}\right)=r\left(\text{i}\right)*\text{cos}\left(\theta \left(\text{i}\right)\right)$$

7 $$\theta \left(i\right)= a * \pi * r$$

8 $$r\left(i\right) =\theta \left(i\right)+ R * rand$$

where $a$ is a parameter that takes a value between 5 and 10 for determining the corner between point search in the central point and $R$ takes a value between 0.5 and 2 for determining the number of search cycles. Third stage is swooping, where bald eagles swing from the best position in the search space to their target prey. All points also move towards the best point. The following equation mathematically illustrates this behaviour.

9 $${P}_{new,i}={rand* P}_{be{st}^{+}}+x1\left(i\right)*\left({P}_{i}-c1*{P}_{mean}\right)+y1(i)({P}_{i}-c2*{\text{P}}_{best})$$

10 $$x1\left(\text{i}\right)=\frac{{x}_{r}\left(\text{i}\right)}{\text{max}\left|xr\right|},y1\left(\text{i}\right)=\frac{{y}_{\Gamma }\left(\text{i}\right)}{\text{max}\left|yr\right|}$$

11 $$xr\left(\text{i}\right)=r\left(\text{i}\right)*\text{sinh}\left(\theta \left(\text{i}\right)\right) , yr\left(\text{i}\right)=r\left(\text{i}\right)*\text{cosh}\left(\theta \left(\text{i}\right)\right)$$

12 $$\theta \left(i\right)= a * \pi * r, r\left(i\right) =\theta \left(i\right)$$

where c1, c2 ∈^1,2^

### Artificial gorilla troops optimizer (GTO)

Artificial Gorilla Troops Optimizer (GTO), established in 2021. In the GTO algorithm five different operators are used for optimization operations (exploration and exploitation) simulated based on gorilla behaviours. We used three different mechanisms for the exploration phase, that is, migration to an unknown location, migration towards a known location, and moving to other gorillas. Each of these three mechanisms is selected according to a general procedure. Three mechanisms can be simulated by the following equation:

13 $$GX\left( {t + 1} \right) = \left\{ {\begin{array}{*{20}l} {\left( {UB - LB} \right) \times r_{1} + LB} & {rand < p,} \\ {\left( {r_{2} - C} \right) \times X_{r} \left( t \right) + L \times H} & {rand \ge 0.5,} \\ {X\left( i \right) - L \times (L \times \left( {X\left( t \right) - GX_{r} \left( t \right)) + r_{3} \times \left( {X\left( t \right) - GX_{r} \left( t \right)} \right)} \right)} & { rand < 0.5,} \\ \end{array} } \right.$$

where $GX\left(t+1\right)$ is the gorilla candidate position vector in the next t iteration. $UB$ and $LB$ represent the upper and lower bounds of the variables, respectively. ${r}_{1}$, ${r}_{2}$, ${r}_{3}$ and $rand$ is random values ranging from 0 to 1 updated in each iteration. $p$ is a parameter that must be given a value before the optimization operation and has a range of 0 – 1. $X\left(t\right)$ is the current vector of the gorilla position. ${X}_{r}\left(t\right)$ is one member of the gorillas in the group randomly selected from the entire population and $G{X}_{r}\left(t\right)$.One of the vectors of gorilla candidate positions randomly selected and includes the positions updated in each phase. $C$, $L$, and $H$ are calculated using the following equations:

14 $$C=F\times \left(1-\frac{It}{MaxIt}\right),$$

15 $$F=\text{cos}\left(2\times {r}_{4}\right)+1,$$

16 $$L=C\times l$$

17 $$\begin{aligned} H =\, & Z \times X\left( t \right), \\ z = & \left[ { - C, C} \right] \\ \end{aligned}$$

In Eq. (14) It is the current iteration value, $MaxIt$ is the total value of iterations, l is a random value in the range of − 1 and 1, ${r}_{4}$ is random value ranging from 0 to 1. In the GTO algorithm’s exploitation phase, has two behaviours of Follow the silverback and Competition for adult females are applied. Follow the silverback can be simulated by this Eq.

18 $$GX\left(t+1\right)=L\times M\times \left(x\left(t\right)-{X}_{silverback}\right)+X\left(t\right),$$

19 $$M={\left({ \left|\frac{1}{N}\sum_{i=1}^{N}G{X}_{i}\left(t\right)\right|}^{g} \right)}^\frac{1}{g}$$

20 $$g= {2}^{L}$$

In Eq. (18), $X\left(t\right)$ is the gorilla position vector, and X_silverback_ is the silverback gorilla position vector (best solution). $G{X}_{i}\left(t\right)$ Eq. (19) shows each candidate gorilla’s vector position in iteration *t.* N represents the total number of gorillas. $L$ is calculated using Eq. (16).

While Competition for adult females can be simulated using the following Eq.

21 $$GX\left(i\right)={X}_{silverback}-\left({X}_{silverback}\times Q-X\left(t\right)\times Q\right)\times A,$$

22 $$Q=2 \times {r}_{5}-1$$

23 $$\begin{aligned} A =\, & \beta { } \times E, \\ E = & \left\{ {\begin{array}{*{20}c} {N_{1} ,} & {rand \ge 0.5,} \\ {N_{2} ,} & {rand \ge 0.5,} \\ \end{array} } \right. \\ \end{aligned}$$

where ${r}_{5}$ is random values ranging from 0 to 1, $\beta$ is a parameter to be given value before the optimization operation. the $E$ 's value of $E$ will be equal to random values in the normal distribution and the problem’s dimensions, but if rand < 0.5, $E$ will be equal to a random value in the normal distribution. rand is also a random value between 0 and 1.

### Particle swarm algorithm (PSO)

It is well-known PSO is an effective metaheuristics algorithm, which has been widely used in different engineering domains^28^ . It mimics the preying behaviour of bird’s folk. In PSO, the movement of each particle is determined by the experiences of the particle itself and those of swarm individuals. Starting from a random population size N, the vector ${d}_{i}$ with ND-dimensional design variables is the ith solution. The new position and population velocity are evaluated using the following formulas.

24 $${V}_{ij}\left(t+1\right)=\omega {V}_{ij}\left(t\right)+{r}_{1ij}{C}_{1}\left({P}_{ji}-{d}_{ij}\left(t\right)\right)+{r}_{2ij}{C}_{2}({P}_{gi}-{d}_{ij}\left(t\right))$$

25 $${d}_{ij}\left(t+1\right)={d}_{ij}\left(t\right)+{v}_{ij}\left(t+1\right)$$

In Eq. (24) ${V}_{ij}\left(t+1\right)$ is the updated velocity. ${V}_{ij}\left(t\right)$ is the current velocity. ${\text{P}}_{\text{ji}}$ is the best position. ${d}_{ij}\left(t\right)$ is the current position. ${r}_{1ij}$ and ${r}_{2ij}$ are two random values lie in [0,1]. The parameters ${C}_{1}$ and ${C}_{2}$ are the stochastic weighting. $\omega$ Controls the impact of previous velocity on the current velocity. Typically, within the range [0, 1]. In Eq. (25) ${d}_{ij}\left(t+1\right)$ is the updated position.

### Ant lion optimizer algorithm (ALO)

Inspired by the hunting behaviour from ant lions^22^. It is a new nature-inspired intelligent technique. The ant lions dig a cone-shaped pit in the sand, in which the trap size is determined by the relations between the hunger level and the moon shape. ALO uses the elitism strategy to update the movements of the ant lions; each ant walks around in the vicinity of a selected ant lion according to the roulette wheel and the elite.

26 $$An{t}_{i}^{t}=\frac{{R}_{A}^{t}+{R}_{E}^{t}}{2}$$

where t denotes the iterative number, $i$ denotes the ant number, ${R}_{A}^{t}$ denotes the walk of ant lion that is determined through the roulette wheel, and ${R}_{E}^{t}$ denotes the random of elite. $An{t}_{i}^{t}$ denotes the position.

### Grey wolf optimizer (GWO)

Mirjalili et al.^29^ recently created the GWO algorithm, in which the hunting information is shared by a grey wolf family. Inspired by the strict social dominant hierarchy, the grey wolf can be divided into three types: alpha (leader), beta (the best candidate), and omega (scapegoat). Their positions can be calculated by.

27 $$\overrightarrow{{d}_{1}}=\overrightarrow{{d}_{\alpha }}-\overrightarrow{{A}_{1}}\left(\overrightarrow{{D}_{\alpha }}\right), \overrightarrow{{d}_{2}}=\overrightarrow{{d}_{\beta }}-\overrightarrow{{A}_{2}}\left(\overrightarrow{{D}_{\beta }}\right) ,\overrightarrow{{d}_{3}}=\overrightarrow{{d}_{\delta }}-\overrightarrow{{A}_{3}}\cdot \left(\overrightarrow{{D}_{\delta }}\right)$$

In Eq. (27) $\overrightarrow{{d}_{1}}, \overrightarrow{{d}_{2}}$, $\overrightarrow{{d}_{3}}$ are updated position. $\overrightarrow{{d}_{\alpha }} , \overrightarrow{{d}_{\beta } } \overrightarrow{,{d}_{\delta }}$ are positions of alpha, beta, gamma wolves. $\overrightarrow{{A}_{1}}$, $\overrightarrow{{A}_{2}}$, $\overrightarrow{{A}_{3}}$ are coefficient vectors. $\overrightarrow{{D}_{\alpha }}$, $\overrightarrow{{D}_{\beta }} , \overrightarrow{{D}_{\delta }}$ are distances between wolves (alpha, beta, gamma) and current positions multiplying by coefficient vectors.

### Harris’s hawk optimizer (HHO)

The HHO, and it uses the surprise pounce behaviour of Harris hawks to mimic the exploratory and exploitative phases of optimization^25^ The Harris hawks track the rabbit using their powerful eyes after patiently waiting. It perches on the tall tree according to other family member positions and the rabbit.

28 $${\text{d}}\left( {t + 1} \right) = \left\{ {\begin{array}{*{20}l} {d_{rand} \left( t \right) - r_{1} \left| {d_{rand} \left( t \right) - 2r_{2} {\text{d}}\left( t \right)} \right|} & {q \ge 0.5} \\ {d_{rabbit} \left( t \right) - d_{m} \left( t \right)) - r_{3} \left( {LB + r_{4} \left( {UB - LB} \right)} \right)} & {q < 0.5} \\ \end{array} } \right.$$

where $d\left(t+1\right)$ is the position vector of hawks in the next iteration $t$, ${d}_{rabbit}(t)$ is the position of rabbit, $d\left(t\right)$ is the current position vector of hawks, ${r}_{1}$, ${r}_{2}$, ${r}_{3}$, ${r}_{4}$, and $q$ are random numbers inside (0,1), which are updated in each iteration, $LB$ and $UB$ show the upper and lower bounds of variables, ${d}_{rand}\left(t\right)$ is a randomly selected hawk from the current population, and ${d}_{m}\left(t\right)$ is the average.

### Tuna swarm optimization (TSO)

TSO is based on the cooperative foraging behaviour of tuna swarm. The work mimics two foraging behaviours of tuna swarm, including spiral foraging that tuna are feeding, they swim by forming a spiral formation to drive their prey into shallow water where they can be attacked more easily which mathematical model in Eq. (29) and parabolic foraging Each tuna swims after the previous individual, forming a parabolic shape to enclose its prey which mathematical model in Eq. (30), Tuna successfully forage by the above two methods. More explanations about TSO can be found in^23^.

29 $${x}_{i}^{t+1}\left\{\begin{array}{l}{\alpha }_{1}({x}_{\text{ran}d}^{t}+\beta \cdot \left|{x}_{\text{ran}d}^{t}-{x}_{i}^{t}\right|+{\alpha }_{2}\cdot {x}_{i}^{t},i=1,\\ {\alpha }_{1}\cdot \left({x}_{rand}^{t}+\beta \cdot \left|{x}_{\text{ran}d}^{t}-{x}_{i}^{t}\right|\right)+{\alpha }_{2}\cdot {X}_{i-1}^{t},i=\text{2,3},\dots ,NP,\\ {\alpha }_{1}\left({x}_{best}^{t}+\beta \cdot \left|{x}_{best}^{t}-{x}_{i}^{t}\right|\right)+{\alpha }_{2}\cdot {x}_{{i}{\prime}}^{t}i=1,\\ {\alpha }_{1}\cdot \left({x}_{best}^{t}+\beta \cdot \left|{x}_{best}^{t}-{x}_{i}^{t}\right|\right)+{\alpha }_{2}\cdot {X}_{i-1}^{t},i=\text{2,3},\dots ,NP,\end{array}\right. \genfrac{}{}{0pt}{}{ if\, rand<\frac{t}{{t}_{\text{max}}},}{if\, rand\ge \frac{t}{{t}_{\text{max}}},}$$

30 $$x_{i}^{t + 1} = \left\{ {\begin{array}{*{20}c} {x_{{b{\text{e}}st}}^{t} + rand \cdot \left( {x_{{b{\text{e}}st}}^{t} - x_{i}^{t} } \right) + TF + 2 \cdot \left( {x_{{b{\text{e}}st}}^{t} - x_{i}^{t} } \right),} & {if\, rand < 0.5} \\ {TF \cdot \rho^{2} x_{i}^{t} , \rho = \left( {1 - \frac{t}{{t_{{{\text{max}}}} }}} \right)^{{t/t_{max} }} ,} & {if\, rand \ge 0.5} \\ \end{array} } \right.$$

31 $$\beta ={e}^{bl} .\text{ cos}(2\pi b)$$

32 $$l = e^{{3{\text{cos}}\left( {\left( {\left( {\frac{{t_{max} + 1}}{t}} \right) - 1} \right){*}\pi } \right)}} ,$$

where ${x}_{i}^{t+1}$ is the ith individual of the $t+1$ iteration, ${x}_{rand}^{t}$ is a randomly generated reference point in the search space. , ${x}_{best}^{t}$ is the current optimal individual (food), ${\alpha }_{1}$ and ${\alpha }_{2}$ are weight coefficients that control the tendency of individuals to move towards the optimal individual and the previous individual, $\beta$ is a parameter calculated according to Eq. (31) and $l$ using Eq. (32). *t* denotes the number of current iterations, ${t}_{\text{max}}$ is the maximum iterations, and $b$ is a random number uniformly distributed between 0 and 1.

### Dingo optimizer (DOA)

DOA mimics the social behaviour of the Australian dingo dog. The algorithm is inspired by the hunting strategies of dingoes which are attacking by persecution, grouping tactics, and scavenging behaviour 27]. To increment the overall efficiency and performance of this method, three search strategies associated with four rules were formulated in the DOA.

First strategy is group attack. predators often use highly intelligent hunting techniques. Dingoes usually hunt small prey, such as rabbits, individually, but when hunting large prey such as kangaroos, they gather in groups. Dingoes can find the location of the prey and surround it, such as wolves, this behaviour is represented by the following equation:

33 $${\overrightarrow{x}}_{i}\left(t+1\right)={\beta }_{1}\sum_{k=1}^{na}\frac{[\overrightarrow{{\varphi }_{k}\left(t\right)}-\overrightarrow{{x}_{i}} \left(t\right)]}{na}-\overrightarrow{{x}_{*}} \left(t\right),$$

where ${\overrightarrow{x}}_{i}\left(t+1\right)$ is the new position of a search agent , na is a random integer number generated in the interval of [2, SizePop/2], where SizePop is the total size (dingoes that will attack) where ${\varphi }_{k}\left(t\right)$ is a subset of search agent , $X$ is the dingoes population randomly generated, ${x}_{i}$(t) is the current search iteration, and β1 is a random number uniformly generated from interval [-2,2], ${x}_{*}$(t) is the best search agent found from the previous iteration. Second strategy is Persecution. Dingoes usually hunt small prey, which is chased until it is caught individually. The following Eq. models this behaviour:

34 $${\overrightarrow{x}}_{ i} \left(t+1\right)={\overrightarrow{x}}_{*}\left(t\right)+{\beta }_{1}*{e}^{{\beta }_{2}}*\left({\overrightarrow{x}}_{{r}_{1}}\left(t\right)-{\overrightarrow{x}}_{ i}\left(t\right)\right),$$

where ${\beta }_{2}$ is a random number uniformly generated in the interval of [− 1, 1], ${r}_{1}$ is the random number generated in the interval from 1 to the size ofa maximum of search agents (dingoes), and i ≠ ${r}_{1}$.

Third strategy is Scavenger, which behaviour is defined as the action when dingoes find carrion to eat when they are randomly walking in their habitat. The following Eq. models this behaviour:

35 $${\overrightarrow{x}}_{i}\left(t+1\right)=\frac{1}{2}\left[{{e}^{{\beta }_{2}}*\overrightarrow{x} }_{{r}_{1}}\left(t\right)-{\left(-1\right)}^{\sigma }*{\overrightarrow{x}}_{i}\left(t\right)\right],$$

where $$\sigma$$ is a $$a binary number randomly \epsilon \left\{\text{0,1}\right\},$$ and i ≠ $${r}_{1}$$.

Fourth strategy Dingoes’ Survival Rates, when the Australian dingo dog is at risk of extinction due to illegal hunting. The dingoes’ survival rate value is provided by the following equation:

36 $${\overrightarrow{x}}_{i}\left(t\right)={\overrightarrow{x}}_{ *}\left(t\right)+\frac{1}{2}\left[{\overrightarrow{x} }_{{r}_{1}}\left(t\right)-{\left(-1\right)}^{\sigma }*{\overrightarrow{x}}_{{ r}_{2}}\left(t\right)\right],$$

### Tunicate swarm algorithm (TSA)

Is a bioinspired metaheuristic optimization algorithm inspired from the swarm behaviours of tunicates during the foraging process. It was proposed by Kaur et al.^24^ TSA algorithm has been constructed mathematically on two main behaviours of tunicates that are jet propulsion and swarm intelligence. To mathematically model the jet propulsion behaviour, a tunicate should satisfy three conditions first Avoiding the conflicts among search agents is represented by the following equations:

37 $$\overrightarrow{A}= \frac{\overrightarrow{G}}{\overrightarrow{M}}$$

38 $$\overrightarrow{G}= {c}_{2}+{c}_{3}-\overrightarrow{F}$$

39 $$\overrightarrow{F}=2 \cdot {c}_{1}$$

40 $$\overrightarrow{M}=[ {P}_{min}+ {c}_{1}\cdot {P}_{max}- {P}_{min}]$$

$\overrightarrow{A}$ Is employed for the calculation of new search agent position. $\overrightarrow{G}$ is the gravity force and $\overrightarrow{F}$ shows the water flow advection in deep ocean. The variables ${c}_{1},{c}_{2}$ and ${c}_{3}$ are random numbers lie in the range of [0, 1]. $\overrightarrow{M}$ represents the social forces between search agents. ${P}_{min} and {P}_{max}$ represent the initial and subordinate speeds to make social interaction.

Second Movement towards the direction of best neighbour is represented by the following equations:

41 $$\overrightarrow{PD}=\left|\overrightarrow{FS}-rand \left[\text{0,1}\right].\right|,$$

where $\overrightarrow{PD}$ is the distance between the food source and search agent, $\overrightarrow{FS}$ is the position of food source $\overrightarrow{{p}_{p}\left(x\right)}$ indicates the position of tunicate. Third stage is Converge towards the best search agent. It can be mathematically formed as.

42 $$\overrightarrow{{P}_{p}\left(x\right)}=\left\{\begin{array}{c}\overrightarrow{FS}+\overrightarrow{A}.\overrightarrow{ PD} \,if \,rand \left[\text{0,1}\right] \ge 0.5,\\ \overrightarrow{FS}-\overrightarrow{A}.\overrightarrow{ PD} \,if\, rand \left[\text{0,1}\right]<0.5\end{array}\right.$$

$\overrightarrow{{P}_{p}\left(x\right)}$ Is the updated position of tunicate with respect to the position of food source $\overrightarrow{FS}$. Then The swarm behaviour of tunicate can be mathematically formed as

43 $$\overrightarrow{{P}_{p} (x+1)}=\frac{\overrightarrow{{P}_{p}(x)}+\overrightarrow{{P}_{p}(x+1)}}{2+{c}_{1}},$$

$\overrightarrow{{P}_{p} (x+1)}$ The final position would be in a random place, within a cylindrical or cone-shaped, which is defined by the position of tunicate.

### Growth optimizer (GO)

Its main design inspiration originates from the learning and reflection mechanisms of individuals in their growth processes in society. Learning is the process of individuals growing up by acquiring knowledge from the outside world. Reflection is the process of checking the individual’s own deficiencies and adjusting the individual’s learning strategies to help the individual’s growth. Learning phase process as facing the gap between individuals, exploring the causes of these gaps, and learning from them can promote an individual’s growth. It can be mathematically formed as:

44 $${\overrightarrow{{x}_{i}}}^{It+1}=x{\overrightarrow{i}}^{It}+K{\overrightarrow{A}}_{1}+K{\overrightarrow{A}}_{2}+K{\overrightarrow{A}}_{3}+K{\overrightarrow{A}}_{4}$$

where $K{\overrightarrow{A}}_{k}$, (k = 1, 2, 3, 4) is the knowledge acquired by the *ith* individual from the kth group of the gap. Reflection phases an individual should check and make up for deficiencies in all aspects and his knowledge should be retained. For their bad aspects, they should learn from outstanding individuals, while their good aspects should be retained. When the lesson of a certain aspect cannot be remedied, the previous knowledge should be abandoned, and systematic learning should be carried out again. The reflective process of GO is mathematically modelled through Eqs. (45) and (46).

45 $${x}_{ij}^{lt+1}=\left\{\begin{array}{c}\left\{\begin{array}{c}lb+{r}_{4}\times \left(ub-lb\right) if {r}_{3}<AF \\ {x}_{ij}^{It}+{r}_{5}\times \left({R}_{j}-{x}_{ij}^{It}\right) else\end{array}if {r}_{2}-{P}_{3}\right.\\ {x}_{i,j}^{It} else\end{array}\right.$$

46 $$AF=0.01+0.99\times \left(1-\frac{FES}{\text{Max}FEs}\right)$$

where *ub* and *lb* are the upper and lower bounds of the search domain, and ${r}_{2}$, ${r}_{3}$, ${r}_{4}$, and ${r}_{5}$ are uniformly distributed random numbers in the range [0,1]. The value of ${P}_{3}$ controls the probability of reflection and is set to 0.3. The attenuation factor ($AF$) is composed of the current number of evaluations ($FES$) and the maximum number of evaluations ($\text{Max}FEs$). ${R}_{j}$ denotes an individual at the high level, and it serves as a reflective learning guider for the current individual i.

## BES–GO algorithm

In this section, we explain BES–GO structure and steps of implementation. As mentioned above BES has three stages select space, search in space, and swoop each stage repeated iterations times. The purpose of selection BES is the power of the algorithm due to balance between exploration and exploitation. It has a cooperative learning strategy that allows individuals in the population to share information and learn from the best solutions found during the search which improves the overall performance. The second algorithm is the GO algorithm which is a remarkably effective algorithm. It consists of two phases learning phase and reflection phase. It is found reflection phase take time although the final optimal solution is improved a little. So, it suggests that creating a hybrid algorithm takes 3 stages of BES but applies conditions that search in space apace and swoop applied only for the first half of iteration and apply the learning phase also for the first half of iteration with little change in its mechanism to simplify calculations which will reduce implementation time. The following is the pseudo-code of the algorithm.

The pseudocode of BES–GO algorithm.
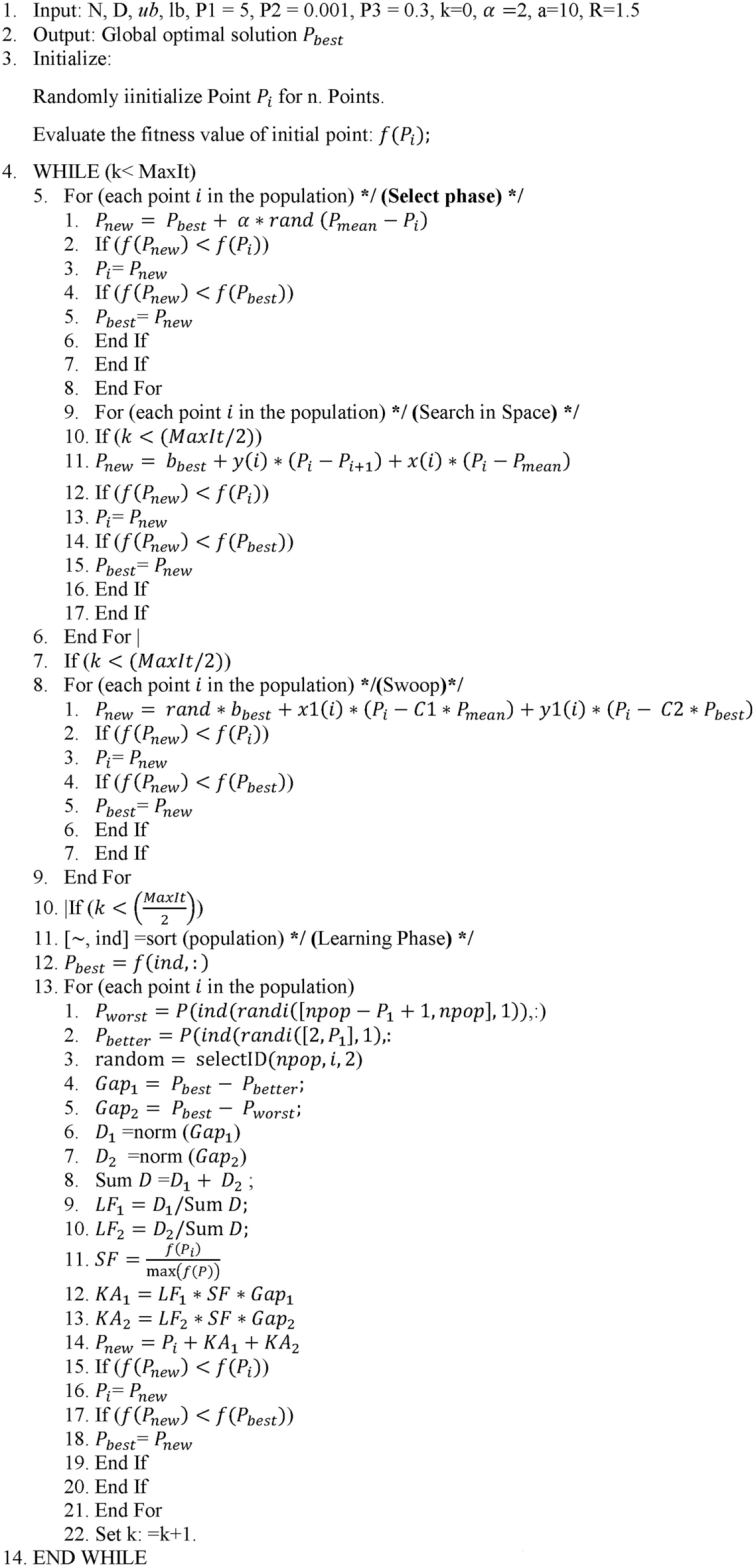


## Engineering structural design problems

In this section, we provide a comprehensive overview of the problems under consideration, encompassing the objective function, decision variables, and constraints. For each problem, we formulate a model and delineate the relevant parameters, alongside presenting the mathematical formulations essential for addressing these problems.

### Welded beam design problem

**Fig. 1:**
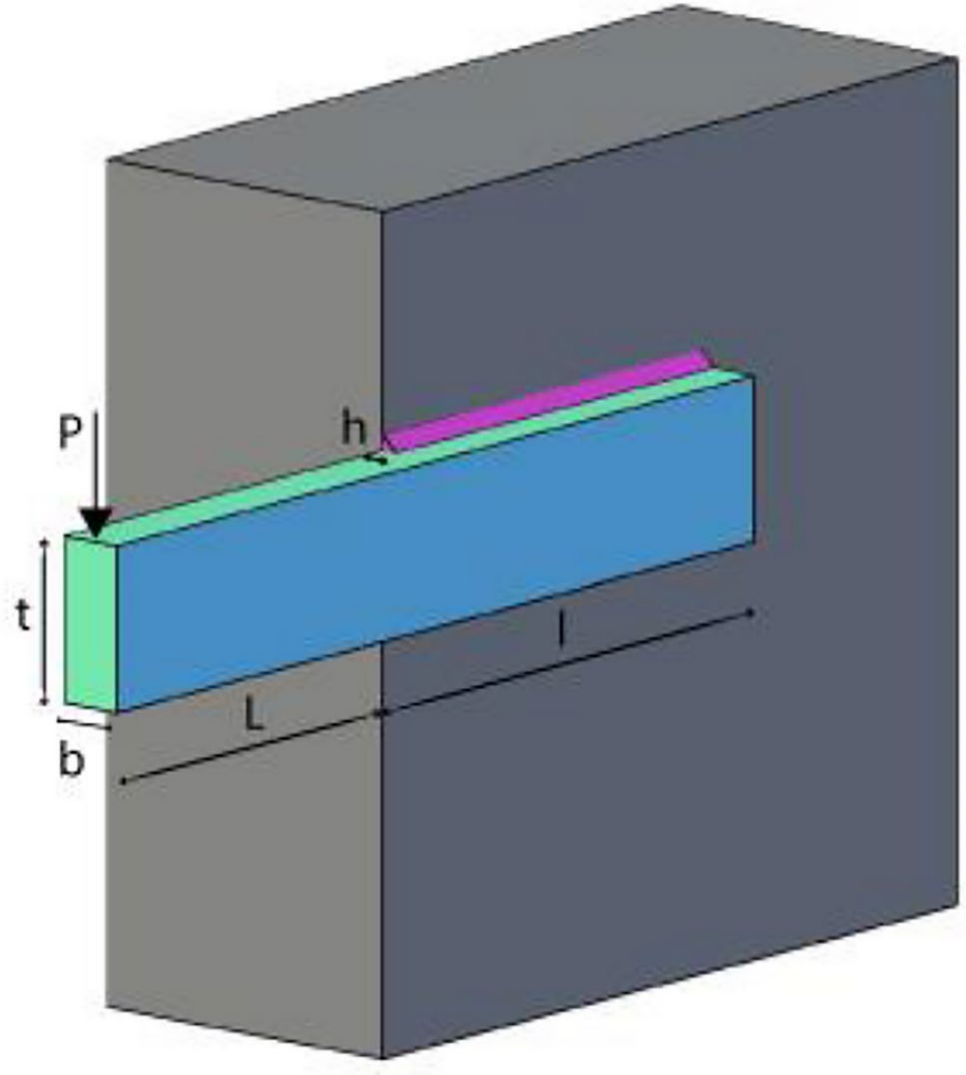
Schematic of the welded beam design problem.

This benchmark problem was introduced by CoelloIn^58^ and many researchers solved it as a real optimization problem. The objective is to minimise the manufacturing cost of the welded beam. It has seven constraints related to deflection, stress, welding, and geometry. The design variables are the weld thickness h (${x}_{1}$), length of the weld l (${x}_{2}$), depth of the beam t (${x}_{3}$), and width of the beam b (${x}_{4}$). Design variables and structure of the welded beam are shown in Fig. 1. The problem can mathematically be stated as:

47 $$\text{Minimize f}\left(\overrightarrow{x}\right)=1.10471{x}_{1}^{2}{x}_{2}+0.04811{x}_{3}{x}_{4}(14+{x}_{2})$$

Subjected to

48 $${g}_{1}\left(\overrightarrow{x}\right)=\tau \left(x\right)-{\tau }_{max}\le 0,$$

49 $${g}_{2}\left(\overrightarrow{x}\right)=\sigma \left(x\right)-{\sigma }_{max}\le 0,$$

50 $${g}_{3}\left(\overrightarrow{x}\right)={x}_{1}-{x}_{4}\le 0,$$

51 $${g}_{4}\left(\overrightarrow{x}\right)=1.10471{x}_{1}^{2}+0.04811{x}_{3}{x}_{4}\left(14+{x}_{2}\right)-5\le 0,$$

52 $${g}_{5}\left(\overrightarrow{x}\right)=0.125-{x}_{1}\le 0,$$

53 $${g}_{6}\left(\overrightarrow{x}\right)=\delta \left(x\right)-{\delta }_{max} \le 0,$$

54 $${g}_{7}\left(\overrightarrow{x}\right)=P-{P}_{c} \le 0,$$

where weld stress τ(x) is calculated using Eq. (55):

55 $$\tau \left( x \right) = \sqrt {\left( {\tau^{\prime } } \right)^{2} + 2\tau^{\prime } \tau^{\prime \prime } \frac{{x_{2} }}{2R} + \left( {\tau^{\prime \prime } } \right)^{2} }$$

56 $$\begin{aligned} \tau^{\prime } = & \frac{P}{{\sqrt {2x_{1} x_{2} } }},{ }\tau^{\prime \prime } = \frac{MR}{J},{\text{ M}} = {\text{P}}\left( {{\text{L}} + \frac{{x_{2} }}{2}} \right).R = \sqrt {\frac{{x_{2}^{2} }}{4} + \left( {\frac{{x_{1} + x_{2} }}{2}} \right)^{2} } \\ J = & 2\left[ {\sqrt 2 x_{1} x_{2} \left\{ {\frac{{x_{2}^{2} }}{12} + \left( {\frac{{x_{1} + x_{3} }}{2}} \right)^{2} } \right\}} \right] \\ \end{aligned}$$

The bar displacement is found using the following equation:

57 $$\delta \left(x\right)=\frac{4P{L}^{3}}{E{x}_{3}^{3}{x}_{4}}$$

The bar bending stress σ(x) is calculated using the following equation:

58 $$\sigma \left(x\right)=\frac{6PL}{{x}_{4}{x}_{3}^{2}},$$

The bar buckling load is calculated using the following equation:

59 $${P}_{c}\left(\text{x}\right)=\frac{4.013E\sqrt{\frac{{x}_{3}^{2}{x}_{4}^{6}}{36}}}{{L}^{2}}\left(1-\frac{{x}_{3}}{2L}\sqrt{\frac{E}{4G}} \right)$$

$$\begin{gathered} \sigma _{{max}} \left( {Yield~\;stress} \right) = {\text{3}}0,000\;{\text{psi}} \hfill \\ {\text{E}}\left( {{\text{Modulus of elasticity}}} \right)\, = \,{\text{3}}0*{\text{1}}0{\text{6 psi}}, \hfill \\ {\text{p}}\left( {{\text{Load}}} \right) = {\text{ 6}}000{\text{ lb}},{\text{ L}}\left( {{\text{Length of beam}}} \right) = {\text{14 in}}, \hfill \\ {\text{G}}({\text{Shearing modulus}}) = {\text{12}}*{\text{1}}0{\text{6 psi}}, \hfill \\ \tau _{{max}} \left( {Design~stress~of~the~weld} \right) = 13,600~{\text{psi}}, \hfill \\ \delta _{{max}} \left( {{\text{Maximum deflection}}} \right)\, = \,0.{\text{25 in}}, \hfill \\ \end{gathered}$$

with, $$0.1\le {x}_{1},{x}_{4}\le 2, 0.1\le {x}_{2},{x}_{3}\le 10.$$

### Weight optimization of cantilever beams

**Fig. 2:**
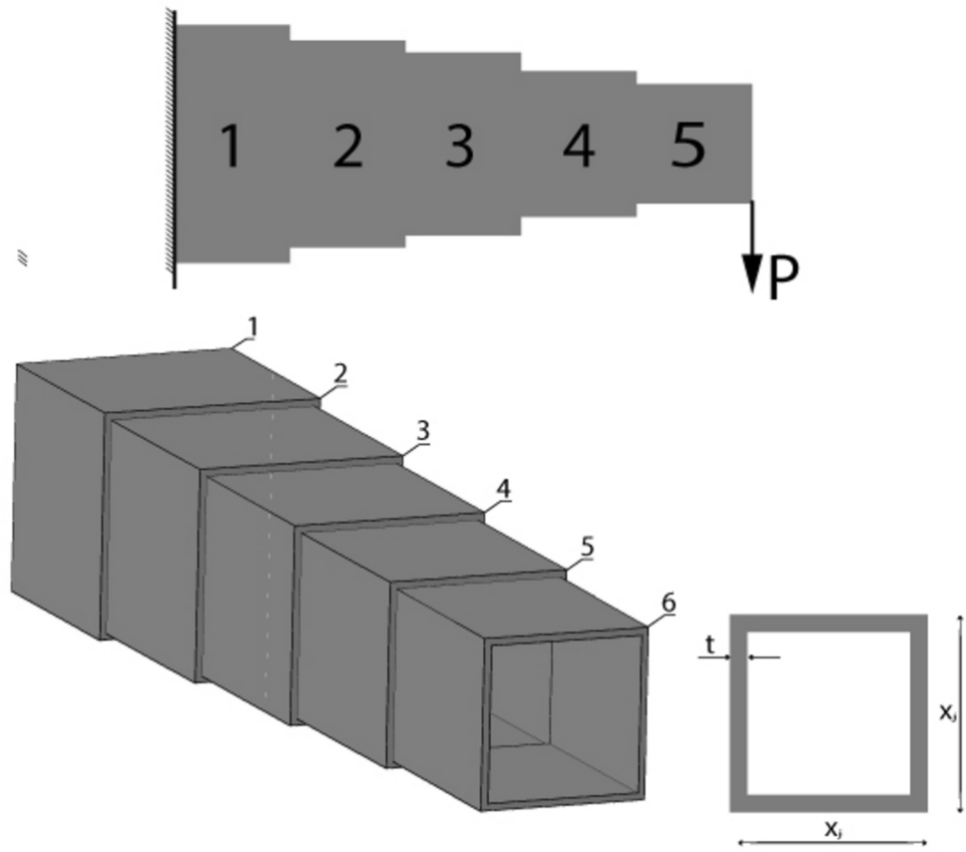
The cantilever beam weight problem.

The second example is weight optimization of cantilever beam shown in Fig. 2 has 5 decision variables which are hollow square cross sections width and height, and the thickness is constant 2/3 for each step. This example was originally given by Fleury and Braibant^59^.The objective function is minimizing the total weight of the beam. The problem can mathematically be stated as.

60 $$Minimize\, f\left(x\right)=0.0624({x}_{1}+{x}_{2}+{x}_{3}+{x}_{4}+{x}_{5})$$

61 $${c}_{1}=\frac{61}{{x}_{1}^{3}}+\frac{37}{{x}_{2}^{3}}+\frac{19}{{x}_{3}^{3}}+\frac{7}{{x}_{4}^{3}}+\frac{1}{{x}_{5}^{3}}\le 0$$

And the solution limits are.

$$0.01{\le x}_{j}\le 100 for j=1 to 5.$$

### I-beam vertical deflection optimization

**Fig. 3:**
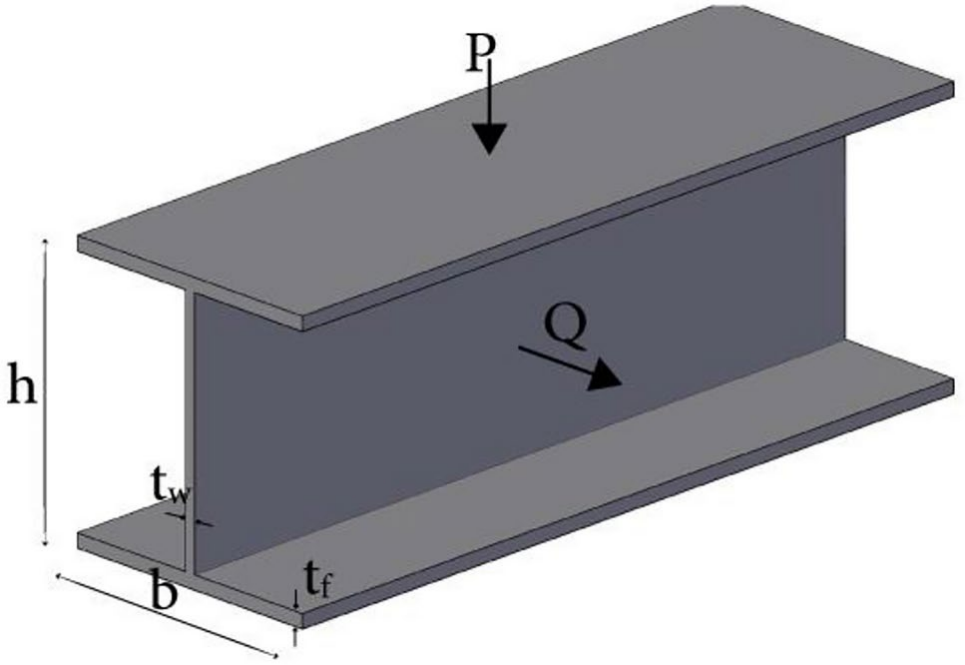
I beam design problem.

This example was originally given by Gold and Krishnamurty^60^. The objective is to minimize the vertical deflection of the I-beam which exposed to vertical design load P and load Q which in another direction which shown in the Fig. 3. Consider the variable x = (${x}_{1}$, ${x}_{2}$, ${x}_{3}$, ${x}_{4}$) = (b, h, ${t}_{w}$, ${t}_{f}$), the mathematical formulation of the problem is defined as follows:

62 $$\text{Minimize }f\left(b,h,{t}_{w},{t}_{f}\right)=\frac{5000}{\frac{{t}_{w}{\left(h-2{t}_{f}\right)}^{3}}{12}+\frac{b{f}_{f}^{3}}{6}+2b{t}_{f}{\left(\frac{h-{t}_{t}}{2}\right)}^{2}}$$

63 $$\text{Such that}.{g}_{1}(\text{x})=2b{t}_{f}+{t}_{w}\left(h-2{t}_{f}\right)\le 300$$

64 $${g}_{2}(x)=\frac{180000h}{{t}_{w}{\left(h-2{t}_{f}\right)}^{3}+2b{t}_{w}\left(4{t}_{f}^{2}+3h\left(h-2{t}_{f}\right)\right)}+\frac{15000b}{{t}_{w}^{3}\left(h-2{t}_{f}\right)+2{t}_{w}{b}^{3}}\le 6$$

where*10* ≤ *h* ≤ *80; 10* ≤ *b* ≤ *50; 0.9* ≤$${t}_{w}$$  ≤ *5; 0.9* ≤ $${t}_{f}$$≤ *5.*

### Tubular column design optimization problem

**Fig. 4:**
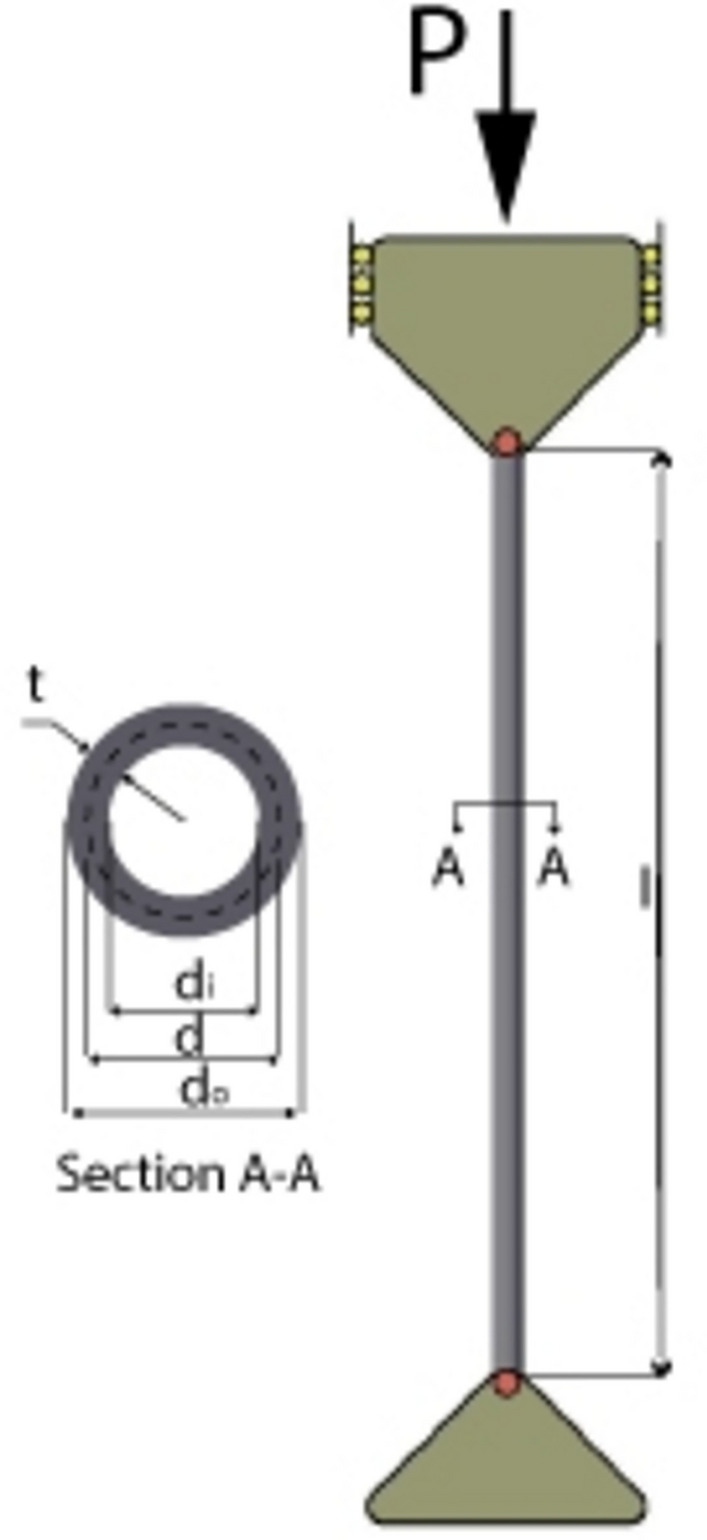
Schematic of the tubular column design problem.

The tubular column^55^ is shown in Fig. 4. The problem is to minimize the cost of the tubular column section subjected to axially loaded with a load (P), and the upper and the lower bounds of the columns are supported by hinged bearings. Consider the variable x = (${x}_{1}$, ${x}_{2}$) = (d, t), the mathematical formulation of the problem is defined as follows:

65 $$\text{Minimize }\text{f}\left(\text{d},\text{t}\right)=9.8*\text{d}*\text{t}+2*\text{d}$$

Subjected to:

66 $${g}_{1}=\frac{P}{\pi dt}\le {\sigma }_{y}$$

67 $${g}_{2}=\text{P}\le \frac{{\pi }^{2}EI}{{(kl)}^{2}}$$

68 $${g}_{3}=\frac{2}{d}-1\le 0$$

69 $${g}_{4}=\frac{\text{d}}{14}-1\le 0$$

70 $${g}_{5}=\frac{0.2}{t}-1\le 0$$

71 $${g}_{6}=\frac{\text{t}}{0.8}-1\le 0$$

where σy (Yield stress) = 500 kg/cm^2^, E (Modulus of elasticity) = 0.85 x 106 kg/cm^2^, ρ(Density)= 0.0025 kg/cm^3^, k = 1 and L (Length of column) =250 cm

$$2\le d\le$$ 14,0 $$.2\le t\le 0.8$$

### A Three-bar truss system optimization problem

**Fig. 5:**
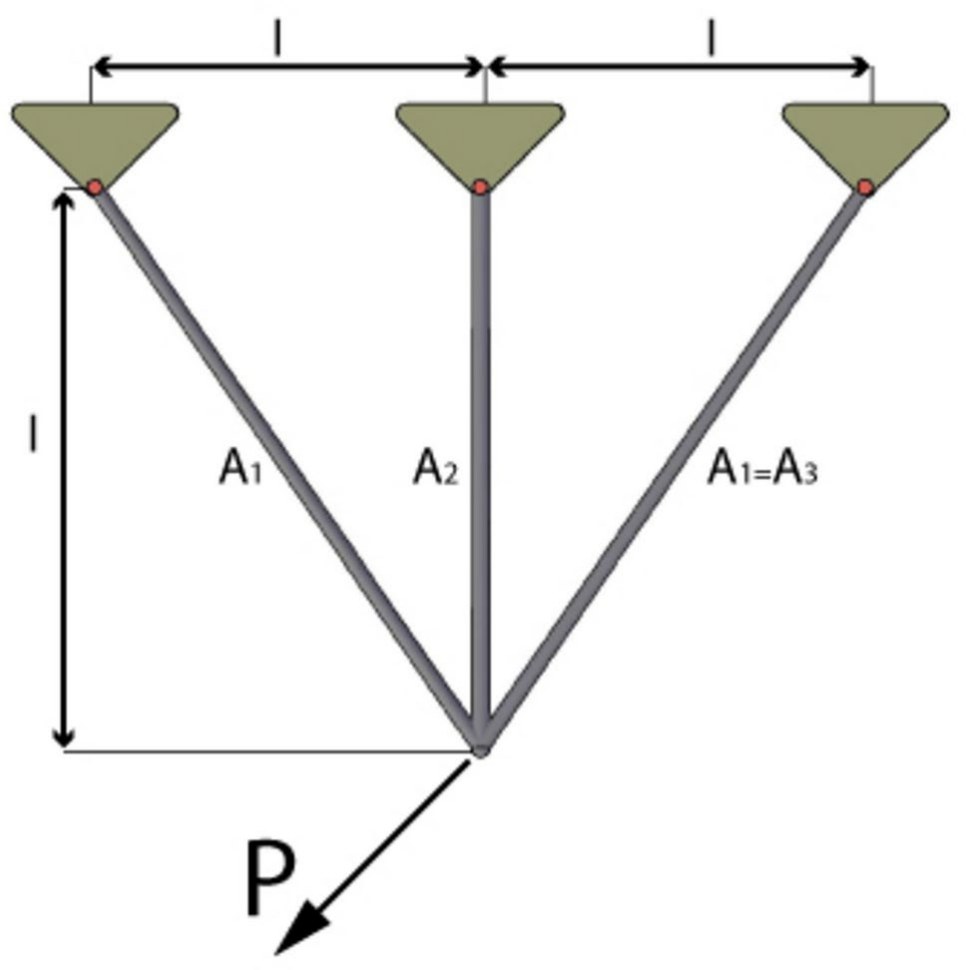
Schematic of the three bar design problem.

A three-bar truss structure is given in Fig. 5. This problem was first presented in Nowcki^21^. This problem requires finding the minimum volume of the truss according to the decision variables of cross-sectional area *x* =  (${x}_{1}$, ${x}_{2}$) = (${A}_{1}$, ${A}_{2}$). The problem is described as follows:

72 $$Minimize :f\left({A}_{1},{A}_{2}\right)=\left(2\sqrt{2}{A}_{1}+{A}_{2}\right)l$$

Subject to:

73 $$g(1)=\frac{\sqrt{2}{A}_{1}+{A}_{2}}{\sqrt{2}{A}_{1}^{2}+2{A}_{1}{A}_{2}}p-\sigma \le 0$$

74 $$g\left(2\right)=\frac{{A}_{2}}{\sqrt{2}{A}_{1}^{2}+2{A}_{l}{A}_{2}}p-\sigma \le 0$$

75 $$g\left(3\right)=\frac{1}{{A}_{1}+\sqrt{2}{A}_{2}}p-\sigma \le 0$$

where 0 ≤$${A}_{1}$$  ≤ 1 and 0 ≤ $${A}_{2}$$  ≤ 1.

l = 100 cm; P = 2 kN, and σ = 2 kN/cm^2^.

## Experimental results analysis

In this section, the results of the numerical investigations are presented in which the capability of the proposed BES–GO algorithm is verified through some mathematical test functions, benchmark suits and some of the well-known engineering design problems.

### Tests of benchmark mathematical functions

BES–GO efficiency is assessed at the IEEE Congress on Evolutionary Computation 2020 (CEC’20)^61^.The simulation results of the proposed BES–GO are compared to other metaheuristics algorithms. To conduct a fair comparison, the BES–GO algorithm and the other competitors are investigated through 30 runs. The number of iterations is set to 500 for all considered problems. Table 6 shows the parameters’ settings for each algorithm.

**Table 6:** Parametrization of BES–GO and the other algorithms.

Algorithm	Parameter
BES–GO	$\alpha =$ 2, a = 10, R = 1.5, P1 = 5, P2 = 0.001, P3 = 0.3
BES	$\alpha =$ 2, a = 10, R = 1.5
GO	P1 = 5, P2 = 0.001, P3 = 0.3
GWO	–
PSO	W = 0.8., C1 = 1.5, C2 = 1.5
TSA	${P}_{min}=1$,${P}_{max}=4$
TSO	aa = 0.7, z = 0.05
DOA	P = 0.5., Q = 0.7
ALO	–
GTO	Β = 3, W = 0.8, *p* = 0.03
HHO	E0 = 1.67, E1 = 1

The algorithm results on the CEC’20 functions are compared. In particular, the efficiency of each algorithm is measured with the average of the best solutions obtained at each run and the corresponding standard deviation (STD). Table 7 presents the average and STD values of each algorithm for functions of 10-dimension, i.e., Dim = 10. The best results are shown in boldface.

**Table 7:** Best, average, and standard deviation of obtained by the algorithms on the CEC’20 test suit with D = 10.

Algorithms		BES_GO	GO	BES	TSO	DOA	ALO	PSO	TSA	GWO	GTO	HHO
F measure												
f1	BEST	1.00E+02	1.02E+02	1.00E+02	1.00E+02	2.95E+07	1.00E+02	1.04E+02	1.21E+08	3.16E+04	1.00E+02	5.31E+05
	Average	1.76E+02	1.43E+03	1.20E+03	2.63E+03	2.58E+09	2.00E+03	1.86E+03	5.04E+09	1.11E+08	3.96E+03	1.33E+06
	STD	1.15E+02	1.48E+03	1.44E+03	2.90E+03	3.65E+09	2.44E+03	2.26E+03	3.95E+09	3.12E+08	3.32E+03	1.22E+06
f2	BEST	1.23E+03	1.27E+03	1.11E+03	1.10E+03	1.70E+03	1.39E+03	1.25E+03	2.04E+03	1.16E+03	1.44E+03	1.58E+03
	Average	1.67E+03	1.69E+03	1.60E+03	1.87E+03	2.63E+03	2.10E+03	1.77E+03	2.63E+03	1.70E+03	2.03E+03	2.02E+03
	STD	2.80E+02	2.78E+02	2.84E+02	3.05E+02	4.53E+02	3.32E+02	3.35E+02	2.75E+02	2.85E+02	2.53E+02	2.63E+02
f3	BEST	7.13E+02	7.18E+02	7.14E+02	7.19E+02	7.33E+02	7.21E+02	7.14E+02	7.83E+02	7.17E+02	7.24E+02	7.40E+02
	Average	7.29E+02	7.39E+02	7.40E+02	7.43E+02	7.79E+02	7.45E+02	7.25E+02	8.04E+02	7.33E+02	7.50E+02	7.92E+02
	STD	8.25E+00	1.40E+01	1.59E+01	1.59E+01	2.57E+01	1.56E+01	9.46E+00	1.05E+01	1.17E+01	1.69E+01	2.43E+01
f4	BEST	1.90E+03	1.90E+03	1.90E+03	1.90E+03	1.90E+03	1.90E+03	1.90E+03	1.92E+03	1.90E+03	1.90E+03	1.90E+03
	Average	1.90E+03	1.90E+03	1.90E+03	1.90E+03	3.83E+03	1.90E+03	1.90E+03	4.99E+04	1.90E+03	1.90E+03	1.91E+03
	STD	5.00E−01	5.97E−01	7.86E−01	2.24E+00	4.01E+03	7.61E−01	3.85E−01	8.11E+04	6.77E+00	1.10E+00	3.68E+00
f5	BEST	1.72E+03	1.75E+03	1.85E+03	2.26E+03	1.96E+03	2.84E+03	2.17E+03	6.52E+04	2.70E+03	1.76E+03	3.86E+03
	Average	2.12E+03	2.28E+03	2.26E+03	7.85E+03	7.82E+04	6.60E+04	5.74E+03	5.40E+05	1.06E+05	2.38E+03	6.88E+04
	STD	2.59E+02	2.92E+02	2.79E+02	8.27E+03	1.84E+05	9.94E+04	4.72E+03	2.47E+05	1.84E+05	3.32E+02	5.66E+04
f6	BEST	1.60E+03	1.60E+03	1.60E+03	1.60E+03	1.60E+03	1.60E+03	1.60E+03	1.60E+03	1.60E+03	1.60E+03	1.60E+03
	Average	1.60E+03	1.61E+03	1.60E+03	1.60E+03	1.62E+03	1.60E+03	1.62E+03	1.63E+03	1.61E+03	1.60E+03	1.62E+03
	STD	1.42E+02	1.69E+02	1.73E+02	1.36E+03	1.65E+05	9.67E+03	1.21E+03	9.31E+04	6.50E+03	2.89E+02	1.16E+05
f7	BEST	2.10E+03	2.10E+03	2.10E+03	2.35E+03	2.27E+03	2.48E+03	2.32E+03	4.97E+03	2.60E+03	2.11E+03	3.33E+03
	Average	2.26E+03	2.31E+03	2.30E+03	3.56E+03	3.65E+04	1.45E+04	3.74E+03	1.06E+05	9.82E+03	2.49E+03	6.51E+04
	STD	1.09E+01	1.50E+01	3.04E+00	3.06E+00	2.23E+01	7.22E+00	1.67E+01	2.33E+01	1.26E+01	2.36E−01	1.28E+01
f8	BEST	2.22E+03	2.30E+03	2.20E+03	2.23E+03	2.24E+03	2.23E+03	2.22E+03	2.34E+03	2.30E+03	2.24E+03	2.29E+03
	Average	2.30E+03	2.30E+03	2.30E+03	2.30E+03	2.53E+03	2.39E+03	2.34E+03	2.82E+03	2.36E+03	2.30E+03	2.45E+03
	STD	1.46E+01	1.02E+00	1.89E+01	1.46E+01	2.44E+02	2.93E+02	2.48E+02	5.81E+02	2.94E+02	1.63E+01	4.19E+02
f9	BEST	2.50E+03	2.50E+03	2.50E+03	2.50E+03	2.59E+03	2.50E+03	2.50E+03	2.59E+03	2.73E+03	2.50E+03	2.50E+03
	Average	2.73E+03	2.74E+03	2.73E+03	2.69E+03	2.78E+03	2.73E+03	2.72E+03	2.85E+03	2.75E+03	2.72E+03	2.79E+03
	STD	4.44E+01	4.51E+01	4.52E+01	1.17E+02	6.93E+01	7.88E+01	8.82E+01	9.21E+01	1.45E+01	9.93E+01	1.20E+02
f10	BEST	2.90E+03	2.90E+03	2.60E+03	2.90E+03	2.93E+03	2.90E+03	2.60E+03	2.92E+03	2.90E+03	2.90E+03	2.90E+03
	Average	2.93E+03	2.93E+03	2.92E+03	2.93E+03	3.06E+03	2.93E+03	2.91E+03	3.18E+03	2.94E+03	2.93E+03	2.93E+03
	STD	2.30E+01	2.25E+01	6.44E+01	2.47E+01	1.04E+02	2.33E+01	6.31E+01	2.11E+02	1.54E+01	2.50E+01	2.31E+01

As shown in Table 7, the BES–GO algorithm demonstrates superior performance across multiple benchmark functions from the CEC2020 suite, consistently outperforming several well-known metaheuristic algorithms. For instance, in F1, BES–GO achieved the best fitness value of 1.0000E+02, outperforming other competitive algorithms such as GO (1.0199E+02), BES (1.0032E+02), and PSO (1.0418E+02). This indicates BES–GO’s ability to achieve high precision in complex optimization problems. In Benchmark 2, it maintains a strong fitness value of 1.2336E+03, closely competing with algorithms like TSO and BES, while outperforming algorithms such as ALO and TSA. Notably, in more challenging functions such as Benchmark 5, BES–GO demonstrates significant superiority over other algorithms, whereas weaker algorithms like TSA (6.5187E+04) and DOA (2.9514E+07) show much higher error values.

**Fig. 6:**
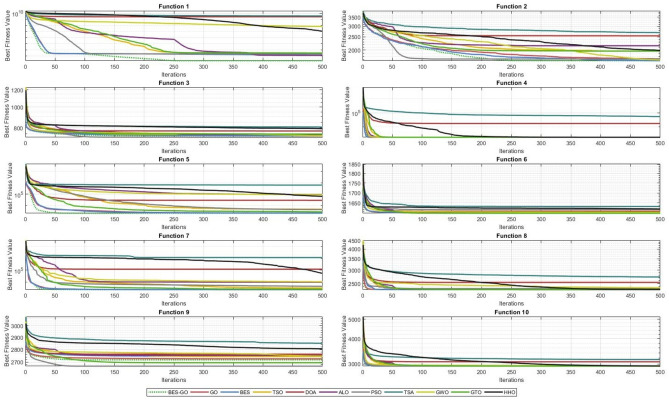
Convergence curves of the proposed BES–GO and the other algorithms obtained on the CEC’20 test suite with Dim = 10.

This consistent performance across benchmarks highlights BES–GO’s effectiveness in balancing exploration and exploitation, adapting well to various problem landscapes. Its stability and precision make it a leading algorithm compared to alternatives like ALO, GWO, GTO, and HHO, which often display higher variability and less competitive results. Overall, BES–GO establishes itself as one of the top-performing algorithms in solving CEC2020 problems, offering enhanced reliability and accuracy in global optimization tasks. The convergence of the algorithms is evaluated (see Fig. 6. As shown in the figure, the BES–GO algorithm converges to (near)-optimal solutions faster than the other algorithms at F1, F3, F4, F5, and F7.

### Structural engineering design problems

These problems are the welded beam, tubular column, cantilever beam weight, three-bar truss system, and I-beam vertical deflection. We have described the mathematical details of these problems in a previous section. Since these problems have constraints, we deal with them using an exterior penalty approach.

We convert equality constraints into inequalities and set a tolerance parameter to 0.0001. We run experiments with a population size of 30 and 500 iterations. Table 6 in the previous section shows the algorithm parameter settings.

To compare how quickly and effectively the algorithms converge, we plot convergence curves showing the history of the best fitness values obtained for each problem. Each algorithm undergoes 30 independent experiments. During these experiments, we record several statistics including the best, mean, worst, and standard deviation of fitness values. The computer used for these experiments has a processor 12th Gen Intel(R) Core (TM) i5-12600KF 3.70 GHz, 32 GB of RAM, and operates on a 64-bit Windows 11 system.

#### The welded beam design problem

**Fig. 7:**
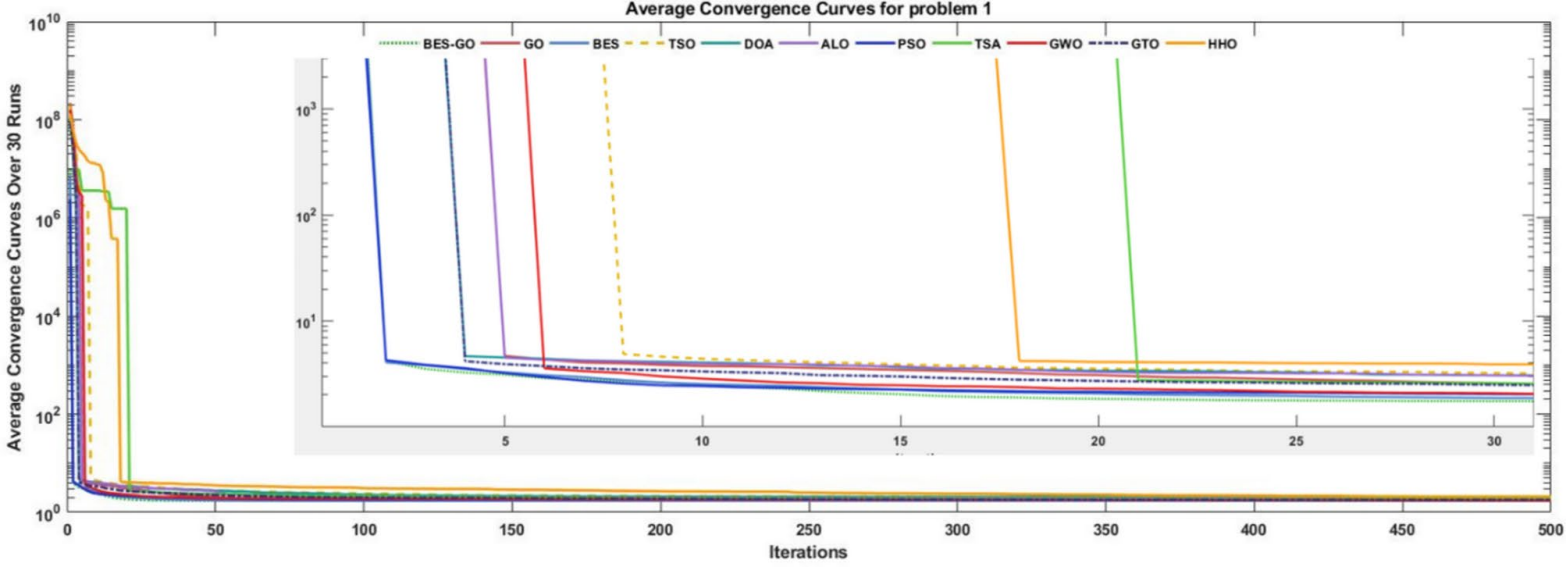
convergence curve for welded beam design problem.

As can be seen from Table 8, The BES–GO algorithm has superior performance in terms of both accuracy and stability compared to other algorithms. It achieves the optimal average and best solution (1.72), with an extremely low standard deviation (7.92E−16), indicating high reliability and minimal variation across runs. Its execution time (5.03E−01) is competitive, and it outperforms others in maintaining a robust worst-case solution (1.72). This balance between computational efficiency and solution quality makes BES–GO an ideal choice for optimization tasks requiring both precision and consistency. The zoomed-in section of the convergence graph, shown in Fig. 7, illustrates that the BES–GO algorithm converges to the optimal solution faster than the other algorithms after only 15 iterations.

**Table 8:** Comparative analysis of various algorithms for the welded beam design problem.

Algorithm	Time	Average	STD	Best	Worst	Solution			
						h	l	t	b
BES–GO	5.03E−01	1.72E+00	**7.92E−16**	1.72E+00	1.72E+00	2.06E−01	3.47E+00	9.04E+00	2.06E−01
BES	6.34E−01	1.72E+00	1.19E−15	1.72E+00	1.72E+00	2.06E−01	3.47E+00	9.04E+00	2.06E−01
GO	3.14E−01	1.72E+00	1.74E−13	1.72E+00	1.72E+00	2.06E−01	3.47E+00	9.04E+00	2.06E−01
GWO	8.90E−02	1.73E+00	2.94E−03	1.73E+00	1.74E+00	2.06E−01	3.48E+00	9.04E+00	2.06E−01
PSO	2.86E−01	1.79E+00	1.02E−01	1.72E+00	2.08E+00	2.06E−01	3.47E+00	9.04E+00	2.06E−01
ALO	1.34E+00	1.84E+00	1.34E−01	1.72E+00	2.27E+00	2.06E−01	3.47E+00	9.04E+00	2.06E−01
GTO	2.42E−01	1.84E+00	2.64E−01	1.72E+00	2.78E+00	2.06E−01	3.47E+00	9.04E+00	2.06E−01
TSA	9.74E−02	1.85E+00	4.34E−02	1.79E+00	1.96E+00	1.82E−01	4.06E+00	9.25E+00	2.05E−01
DOA	1.31E−01	1.86E+00	2.32E−01	1.72E+00	2.59E+00	2.06E−01	3.47E+00	9.04E+00	2.06E−01
TSO	9.45E−02	1.98E+00	3.10E−01	1.73E+00	2.73E+00	2.06E−01	3.47E+00	9.04E+00	2.06E−01
HHO	2.08E−01	2.21E+00	3.66E−01	1.79E+00	2.98E+00	2.07E−01	3.93E+00	9.01E+00	2.07E−01

**Fig. 8:**
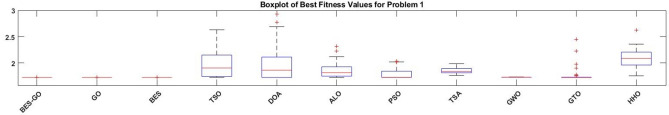
Box plot for welded beam design problem.

Additionally, the box plot in Fig. 8 indicates that the BES–GO, BES, and GO algorithms exhibit superior performance compared to alternative algorithms in terms of performance metrics.

#### The cantilever beam weight optimization problem

**Fig. 9:**
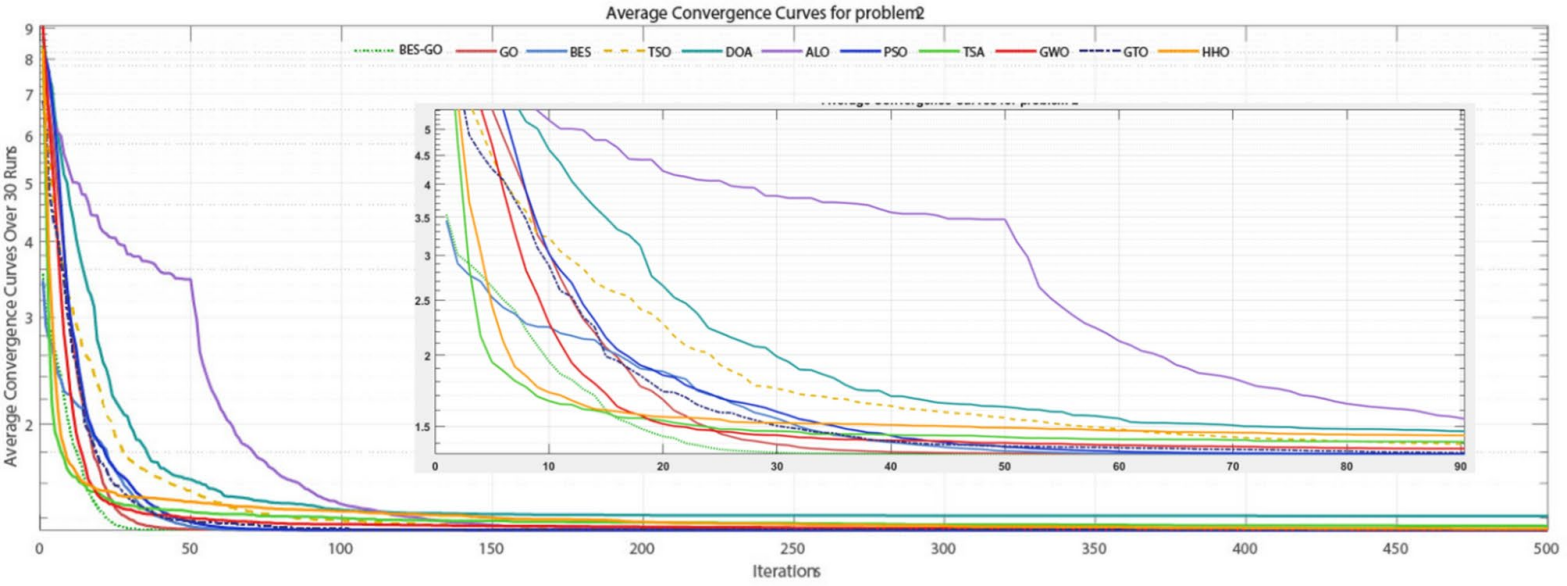
Convergence curve for cantilever beam weight optimization problem.

**Fig. 10:**
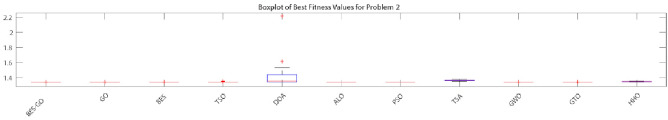
Box plot for Cantilever Beam Weight Optimization problem.

As can be seen from Table 9, The BES–GO algorithm has a quick execution time of 0.431 s and maintains an average value of 1.34. Its standard deviation is remarkably low (4.16E−16), indicating minimal variation in performance across trials (Figs. 8, 9, 10).

**Table 9:** Comparative analysis of various algorithms for the cantilever beam weight optimization problem.

Algorithm	Time	Average	STD	Best	Worst	Solution				
						${x}_{1}$	${x}_{2}$	${x}_{3}$	${x}_{4}$	${x}_{5}$
BES–GO	4.31E−01	1.34E+00	4.16E−16	1.34E+00	1.34E+00	6.02E+00	5.31E+00	4.49E+00	3.50E+00	2.15E+00
GO	2.81E−01	1.34E+00	5.36E−12	1.34E+00	1.34E+00	6.02E+00	5.31E+00	4.49E+00	3.50E+00	2.15E+00
BES	5.42E−01	1.34E+00	1.96E−07	1.34E+00	1.34E+00	6.02E+00	5.31E+00	4.49E+00	3.50E+00	2.15E+00
TSO	6.21E−02	1.34E+00	2.76E−03	1.34E+00	1.35E+00	6.00E+00	5.32E+00	4.49E+00	3.52E+00	2.15E+00
DOA	9.65E−02	1.41E+00	1.68E−01	1.34E+00	2.21E+00	6.02E+00	5.31E+00	4.50E+00	3.50E+00	2.15E+00
ALO	1.63E+00	1.34E+00	1.51E−04	1.34E+00	1.34E+00	6.02E+00	5.32E+00	4.49E+00	3.48E+00	2.16E+00
PSO	2.52E−01	1.34E+00	7.07E−05	1.34E+00	1.34E+00	6.02E+00	5.31E+00	4.49E+00	3.51E+00	2.15E+00
TSA	6.32E−02	1.36E+00	7.50E−03	1.35E+00	1.38E+00	6.30E+00	5.63E+00	4.26E+00	3.40E+00	2.01E+00
GWO	6.23E−02	1.34E+00	1.40E−04	1.34E+00	1.34E+00	6.00E+00	5.32E+00	4.49E+00	3.51E+00	2.15E+00
GTO	1.88E−01	1.34E+00	2.04E−04	1.34E+00	1.34E+00	6.02E+00	5.30E+00	4.49E+00	3.52E+00	2.15E+00
HHO	1.43E−01	1.35E+00	2.99E−03	1.34E+00	1.35E+00	6.17E+00	5.33E+00	4.49E+00	3.36E+00	2.15E+00

The best and worst solution values are consistently at 1.34, reflecting the algorithm’s reliability. In comparison, other algorithms, such as the GO and BES, show similar average solutions but have higher standard deviations, suggesting less stability.

Algorithms like TSO and TSA also perform well, with execution times below 0.1 s, but their average solutions (1.34 for TSO and 1.36 for TSA) are slightly less optimal than those of BES–GO. Overall, BES–GO demonstrates a strong balance of speed, reliability, and consistent performance, making it a commendable choice for optimization tasks.

Additionally, the BES–GO algorithm has a superior convergence curve Fig. 9, reaching the optimal solution after just 20 iterations. This rapid convergence underscores its efficiency in finding high-quality solutions quickly. Moreover, the boxplot in Fig. 10 analysis further confirms BES–GO’s robust performance, showing minimal variation in results compared to other algorithms.

#### I-beam vertical deflection optimization problem

**Fig. 11:**
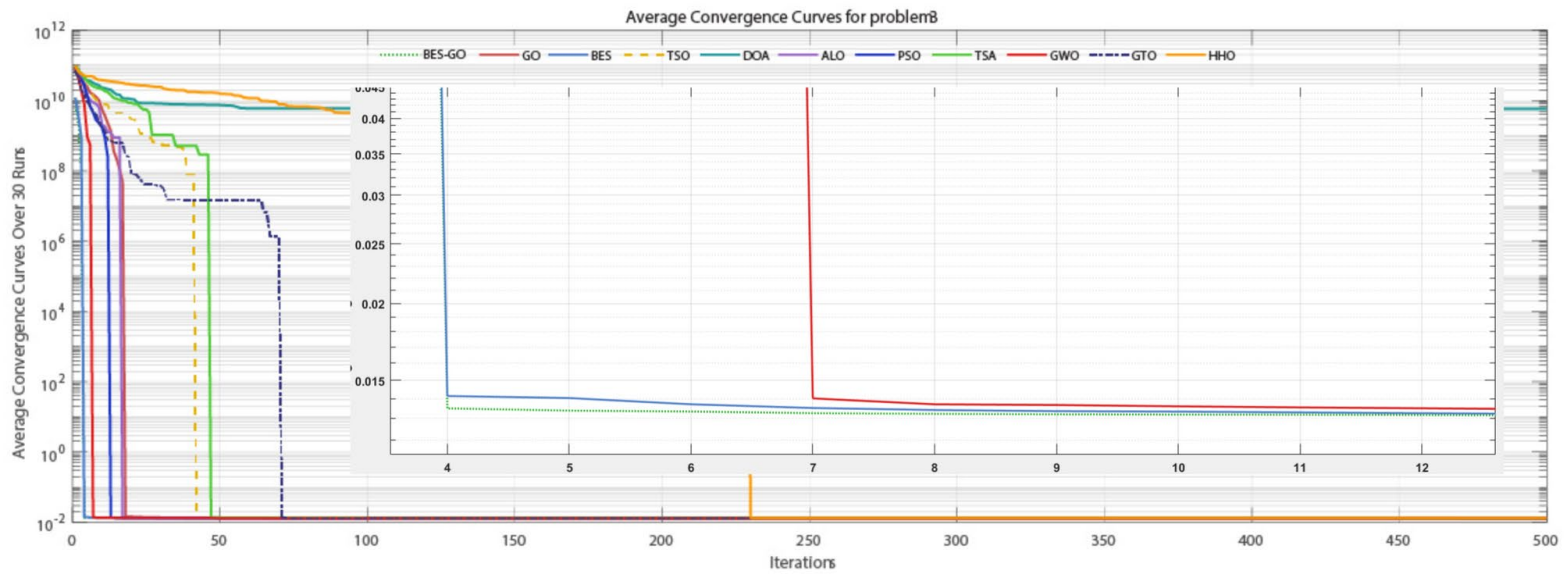
Convergence curve for -Beam Vertical Deflection Optimization problem.

**Fig. 12:**
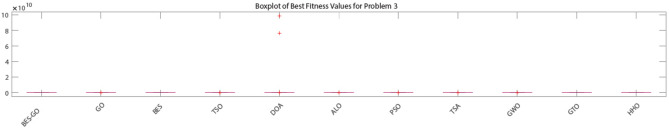
Box plot for Beam Vertical Deflection Optimization problem.

From Table 10 Notably, the BES–GO algorithm demonstrates a commendable performance with a computation time of 0.413 s, achieving an average solution of 1.31E−02 and a remarkably low standard deviation of 8.82E−18, indicating high consistency in its results. The best and worst solutions for BES–GO are both recorded on 1.31E−02, showcasing its reliability in producing optimal outcomes. In comparison, other algorithms like GTO and TSO exhibit faster computation times, 0.159 s, and 0.053 s respectively, yet they maintain similar average and best solution values as BES–GO. This suggests that while some algorithms may operate more quickly, their effectiveness in yielding superior solutions does not surpass that of BES–GO. From Fig. 11, it is observed that BES–GO converge slightly faster to the optimal solution. Furthermore, as depicted in the boxplot in Fig. 12, it is observed that BES–GO have a performance slightly better to other algorithms.

**Table 10:** Comparative analysis of various algorithms for I-beam vertical deflection optimization problem.

Algorithm	Time	Average	STD	Best	Worst	Solution			
						${x}_{1}$	${x}_{2}$	${x}_{3}$	${x}_{4}$
BES–GO	4.13E−01	1.31E−02	8.82E−18	1.31E−02	1.31E−02	5.00E+01	8.00E+01	9.00E−01	2.32E+00
BES	5.18E−01	1.31E−02	8.82E−18	1.31E−02	1.31E−02	5.00E+01	8.00E+01	9.00E−01	2.32E+00
GTO	1.59E−01	1.31E−02	8.82E−18	1.31E−02	1.31E−02	5.00E+01	8.00E+01	9.00E−01	2.32E+00
GO	2.52E−01	1.31E−02	6.89E−12	1.31E−02	1.31E−02	5.00E+01	8.00E+01	9.00E−01	2.32E+00
TSO	5.34E−02	1.31E−02	3.88E−07	1.31E−02	1.31E−02	5.00E+01	8.00E+01	9.00E−01	2.32E+00
GWO	5.38E−02	1.31E−02	6.27E−07	1.31E−02	1.31E−02	5.00E+01	8.00E+01	9.00E−01	2.32E+00
TSA	5.42E−02	1.31E−02	6.22E−05	1.31E−02	1.34E−02	5.00E+01	8.00E+01	9.00E−01	2.32E+00
PSO	2.45E−01	1.31E−02	8.48E−05	1.31E−02	1.34E−02	5.00E+01	8.00E+01	9.00E−01	2.32E+00
ALO	1.35E+00	1.31E−02	1.07E−04	1.31E−02	1.35E−02	5.00E+01	8.00E+01	9.00E−01	2.32E+00
HHO	1.25E−01	1.32E−02	1.66E−04	1.31E−02	1.36E−02	5.00E+01	8.00E+01	9.00E−01	2.32E+00
DOA	8.99E−02	5.84E+09	2.24E+10	1.31E−02	9.86E+10	5.00E+01	8.00E+01	9.00E−01	2.32E+00

#### Tubular column design optimization problem

**Fig. 13:**
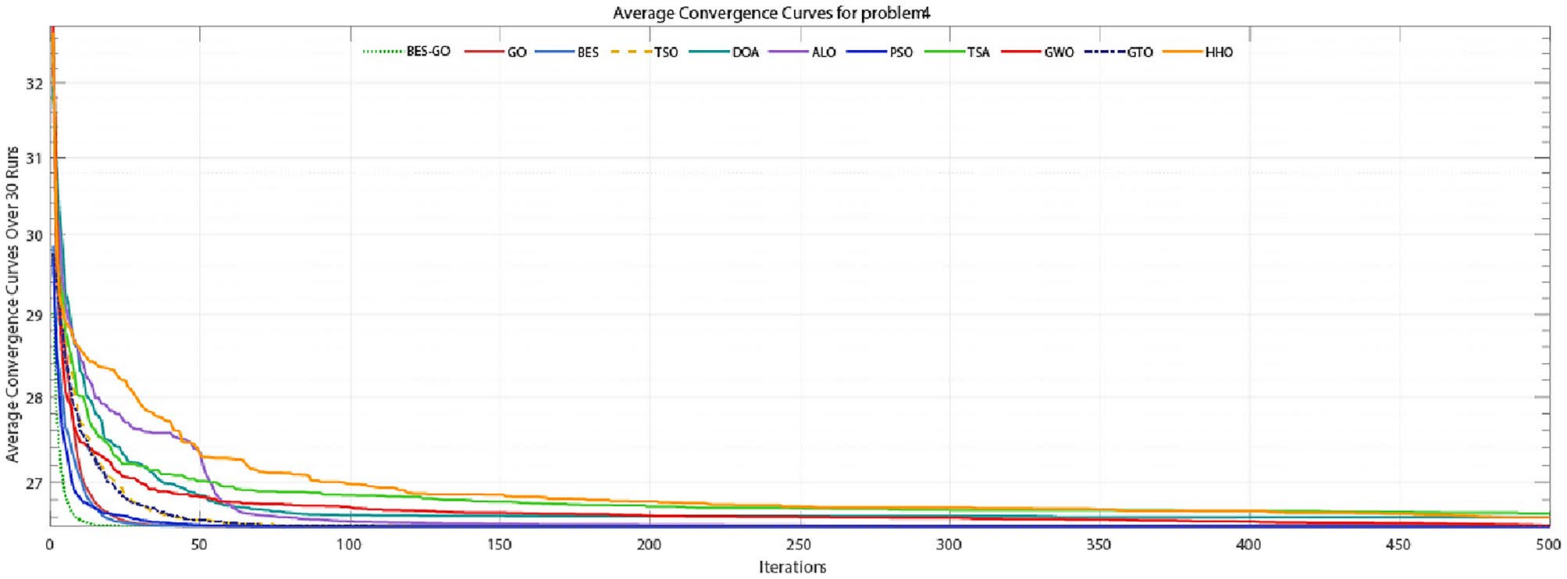
Convergence curve for Tubular Column Design Optimization Problem.

**Fig. 14:**
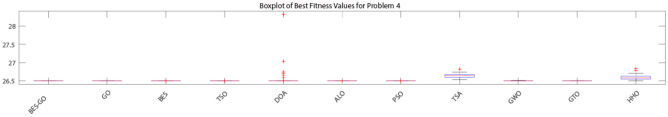
Box plot for Tubular Column Design Optimization Problem.

In Table 11, all algorithms except GWO, HHO, TSA, and DOA demonstrate the best fitness after 30 independent runs, the BES–GO algorithm stands out with a computation time of **0.383** s, achieving an average solution of **26.5** with an incredibly low standard deviation of **3.61E−15**. Furthermore, analysis of Fig. 13 reveals that BES–GO is the fastest algorithm among the alternatives for this problem. Additionally, from Fig. 14, it is apparent that the performance of all algorithms is except HHO, TSA, and DOA closely clustered, indicating similar levels of effectiveness.

**Table 11:** Comparative analysis of various algorithms for the tubular column design optimization problem.

Algorithm	Time	Average	STD	Best	Worst	Solution	
						${x}_{1}$	${x}_{2}$
BES–GO	3.83E−01	2.65E+01	3.61E−15	2.65E+01	2.65E+01	5.45E+00	2.92E−01
GO	1.95E−01	2.65E+01	3.61E−15	2.65E+01	2.65E+01	5.45E+00	2.92E−01
BES	4.92E−01	2.65E+01	3.61E−15	2.65E+01	2.65E+01	5.45E+00	2.92E−01
PSO	2.34E−01	2.65E+01	9.65E−15	2.65E+01	2.65E+01	5.45E+00	2.92E−01
GTO	1.53E−01	2.65E+01	9.28E−15	2.65E+01	2.65E+01	5.45E+00	2.92E−01
TSO	5.14E−02	2.65E+01	2.89E−10	2.65E+01	2.65E+01	5.45E+00	2.92E−01
ALO	6.92E−01	2.65E+01	8.93E−07	2.65E+01	2.65E+01	5.45E+00	2.92E−01
GWO	4.32E−02	2.65E+01	2.03E−03	2.65E+01	2.65E+01	5.45E+00	2.92E−01
HHO	1.10E−01	2.66E+01	7.83E−02	2.65E+01	2.68E+01	5.45E+00	2.92E−01
DOA	8.76E−02	2.66E+01	3.43E−01	2.65E+01	2.83E+01	5.45E+00	2.92E−01
TSA	5.16E−02	2.66E+01	6.31E−02	2.65E+01	2.68E+01	5.46E+00	2.92E−01

#### Three-bar truss system optimization problem

**Fig. 15:**
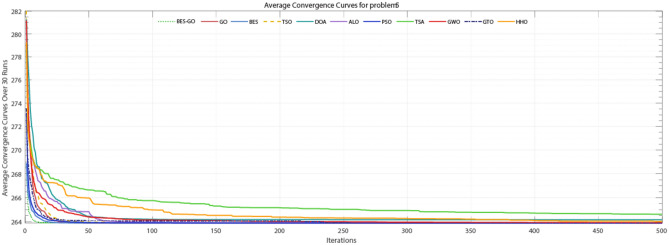
Convergence curve for three-bar truss system optimization problem.

**Fig. 16:**
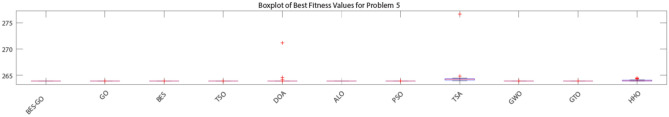
Box plot for three-bar truss system optimization problem.

According to Table 12, the BES–GO algorithm has zero standard deviation indicating exceptional stability across iterations, with no variability in results, which is a strong indicator of reliability. Figure 15, it becomes evident that the BES–GO algorithm is faster than other algorithms when converging to the optimal solution after only a few iterations. Moreover, from Fig. 16, it is apparent that all algorithms demonstrate similar performance levels except TSA, HHO, and DOA.

**Table 12:** Comparative analysis of various algorithms for the three-bar truss system optimization problem.

Algorithm	Time	Average	STD	Best	Worst	Solution	
						${x}_{1}$	${x}_{2}$
BES–GO	4.37E−01	2.64E+02	0.00E+00	2.64E+02	2.64E+02	7.89E−01	4.08E−01
GO	2.42E−01	2.64E+02	1.83E−14	2.64E+02	2.64E+02	7.89E−01	4.08E−01
BES	5.54E−01	2.64E+02	2.36E−14	2.64E+02	2.64E+02	7.89E−01	4.08E−01
GTO	1.91E−01	2.64E+02	1.22E−04	2.64E+02	2.64E+02	7.89E−01	4.08E−01
TSO	6.92E−02	2.64E+02	9.23E−04	2.64E+02	2.64E+02	7.89E−01	4.08E−01
PSO	2.55E−01	2.64E+02	2.46E−03	2.64E+02	2.64E+02	7.89E−01	4.08E−01
ALO	7.09E−01	2.64E+02	3.56E−03	2.64E+02	2.64E+02	7.89E−01	4.09E−01
GWO	5.97E−02	2.64E+02	5.50E−03	2.64E+02	2.64E+02	7.89E−01	4.08E−01
HHO	1.55E−01	2.64E+02	1.68E−01	2.64E+02	2.65E+02	7.88E−01	4.09E−01
DOA	1.02E−01	2.64E+02	1.33E+00	2.64E+02	2.71E+02	7.89E−01	4.08E−01
TSA	6.95E−02	2.65E+02	2.28E+00	2.64E+02	2.77E+02	7.87E−01	4.13E−01

## Nonparametric statistical analysis

In this section, Friedman test^62^ is utilized to assess the performance of metaheuristic algorithms across the five problems. Furthermore, the Wilcoxon signed-rank test^63^ is performed to illustrate the significant differences for BES–GO with the other algorithms.

### Friedman test

The nonparametric Friedman test is used with a significance level of 0.05 to statistically assess the experimental results. The Friedman statistical test is performed to demonstrate whether the differences in performance between the compared algorithms are significant or not in solving the five problems. In the Friedman test, the smaller the ranking, the better the performance of the algorithm. One more important term in the Friedman test is the p-value. A p-value gives an indicator whether there is a significant difference between algorithms or not, where the smaller the p-value, the stronger the evidence of a significant difference. In Friedman’s test statistics in Table 13, SS stands for Sum of Squares refers to the variation among the compared algorithms, df is degrees of freedom, and MS is calculated by dividing the Sum of Squares SS by the degrees of freedom df. Chi-square is a test statistic used to assess the significance of the differences between groups (Table 14).

**Table 13:** Friedman analysis of variance table: MATLAB output.

Source	SS	df	MS	Chi-sq	Prob > Chi-sq
Columns	569.967	10	56.9967	71.84	1.95019e−11
Error	620.033	140	4.4288	-	-
Total	1190	164

**Table 14:** Rank of algorithms according to Fridman test.

Algorithms	BES–GO	GO	BES	TSO	DOA	ALO	PSO	TSA	GWO	GTO	HHO
Average. Rank	3.63	4.97	3.93	5.00	7.43	6.03	4.47	9.83	6.77	5.47	8.47
Rank	1	4	2	5	9	7	3	11	8	6	10

The Probability (Prob > Chi-sq.) is the *p*-value. *P*-value = 1.95019e−11much lower than 0.05 was obtained, which clearly indicates significant differences in terms of precision and computational time.

Table 14 shows that BES–GO achieved the highest rank with an average rank of 3.63. BES and PSO followed in the second and third positions, respectively. GO and TSO are in the fourth and fifth ranks, while GTO and ALO occupy the sixth and seventh positions. GWO is the eighth then DOA. HHO and TSA exhibited the lowest performance, ranking last.

### The Wilcoxon signed-rank test

The Wilcoxon signed-rank test was also performed to detect significant differences between the behaviors of the algorithms’ pair. Wilcoxon test provides the p-value, that is, the probability that the difference in the performance achieved by the two algorithms is obtained by chance and is not statistically significant. In Table 15, BES–GO is compared with the other algorithms in solving the five problems, respectively.

**Table 15:** Wilcoxon test summary for BES with other algorithms.

BES–GO vs Other Algorithms	GO	BES	TSO	DOA	ALO	PSO	TSA	GWO	GTO	HHO
f1	4.97E−05	2.21E−05	4.62E−04	1.78E−06	7.88E−06	9.11E−05	1.78E−06	1.78E−06	4.55E−05	1.78E−06
f2	9.96E−01	3.41E−01	1.41E−03	2.18E−06	5.42E−05	5.07E−01	1.78E−06	8.33E−01	3.01E−03	1.84E−05
f3	4.44E−01	8.17E−01	1.86E−01	2.67E−06	1.42E−02	3.92E−03	1.78E−06	1.93E−01	9.99E−03	1.78E−06
f4	3.85E−01	6.77E−01	6.46E−05	1.78E−06	1.59E−02	6.04E−01	1.78E−06	4.85E−06	6.94E−03	1.78E−06
f5	1.26E−02	3.67E−03	1.78E−06	2.95E−06	1.78E−06	2.67E−06	1.78E−06	1.78E−06	3.13E−04	1.78E−06
f6	5.26E−02	7.07E−01	6.77E−01	2.09E−04	1.68E−05	2.43E−05	1.78E−06	2.27E−04	1.32E−01	1.78E−06
f7	1.86E−01	1.73E−01	2.18E−06	7.15E−06	1.78E−06	2.41E−06	1.78E−06	1.78E−06	2.29E−03	1.78E−06
f8	8.65E−01	4.77E−02	1.67E−01	1.78E−06	1.12E−02	3.31E−01	1.78E−06	3.66E−04	1.22E−01	8.37E−05
f9	9.63E−01	3.52E−01	2.34E−02	6.27E−04	1.34E−02	8.01E−01	1.41E−03	9.41E−03	1.67E−01	3.18E−05
f10	2.92E−01	6.04E−01	3.92E−02	1.97E−06	1.42E−02	7.91E−01	5.35E−06	6.34E−02	2.15E−01	9.41E−03
Problem 01	2.17E−06	6.95E−01	1.78E−06	1.78E−06	1.78E−06	1.78E−06	1.78E−06	1.78E−06	1.78E−06	1.78E−06
Problem 02	1.78E−06	1.78E−06	1.78E−06	1.78E−06	1.78E−06	1.78E−06	1.78E−06	1.78E−06	1.78E−06	1.78E−06
Problem 03	3.13E−02	1	6.10E−05	9.21E−05	1.78E−06	3.90E−06	1.78E−06	1.78E−06	1	8.55E−06
Problem 04	1	1	2.62E−06	1.87E−05	1.78E−06	1.56E−02	1.78E−06	1.78E−06	3.91E−03	1.78E−06
Problem 05	1.00E+00	3.13E−01	1.78E−06	3.16E−06	1.78E−06	1.78E−06	1.78E−06	1.78E−06	2.63E−06	1.78E−06

As shown in Table 15 the Wilcoxon test results indicate that the BES–GO had a better statistically significant performance compared to other algorithms except BES and GO as the p-value is smaller than the level of significance (Alpha = 5%).

## Conclusion and future work

This paper proposes a novel hybrid algorithm called BES–GO, which combines the Bald Eagle Search (BES) algorithm with the Growth Optimizer (GO) algorithm. The BES–GO algorithm is compared against ten other algorithms, including BES, GO, ALO, TSO, TSA, HHO, GTO, DOA, PSO, and GWO, using CEC’20 tests and five benchmark structural design optimization problems. These problems are the vertical deflection of an I-beam, welded beam design, tubular column design, three-bar truss, and cantilever weight optimization. Among all the algorithms, BES–GO consistently achieves the optimal solution for all five problems, demonstrating the smallest standard deviation and a reasonable execution time. This highlights the significant potential of the BES–GO algorithm to advance the field of structural design optimization.

In future work, researchers can make modifications to the BES–GO algorithm to enhance its efficiency and applicability in solving various complex applications within a shorter time. Additionally, developing a multi-objective version of the BES–GO algorithm could enable its utilization in addressing diverse multi-objective optimization problems, including those encountered in tower design.

## Data Availability

The datasets used and/or analysed during the current study are available from the corresponding author on reasonable request.

## References

[1] Remacle, J., Lambrechts, J. & Seny, B. Blossom-Quad: A non-uniform quadrilateral mesh generator using a minimum-cost perfect-matching algorithm. *Int. J. Numer. Methods Eng.* **89**(February), 1102–1119. 10.1002/nme.3279 (2012).

[2] Mortazavi, A. Comparative assessment of five metaheuristic methods on distinct problems. *DÜMF Mühendislik Dergisi* **10**(3), 879–898. 10.24012/dumf.585790 (2019).

[3] Abdel-Basset, M., Abdel-Fatah, L. & Sangaiah, A. K. Metaheuristic algorithms: A comprehensive review In (Elsevier, 2018). 10.1016/B978-0-12-813314-9.00010-4.

[4] Almufti, S. M., Ahmad Shaban, A., Arif Ali, Z., Ismael Ali, R. & Dela Fuente, J. A. Overview of metaheuristic algorithms. *Polaris Global J. Sch. Res. Trends* **2**(2), 10–32. 10.58429/pgjsrt.v2n2a144 (2023).

[5] Mzili, T., Mzili, I. & Riffi, M. E. Artificial rat optimization with decision-making: a bio-inspired metaheuristic algorithm for solving the traveling salesman problem. *Decis. Mak. Appl. Manag. Eng.* **6**(2), 150–176. 10.31181/dmame622023644 (2023).

[6] Abdel-Basset, M. et al. A novel binary Kepler optimization algorithm for 0–1 knapsack problems: Methods and applications. *Alex. Eng. J.* **82**(September), 358–376. 10.1016/j.aej.2023.09.072 (2023).

[7] Munien, C. & Ezugwu, A. E. Metaheuristic algorithms for one-dimensional bin-packing problems: A survey of recent advances and applications. *J. Intell. Syst.* **30**(1), 636–663. 10.1515/jisys-2020-0117 (2021).

[8] Tang, J., Liu, G. & Pan, Q. A review on representative swarm intelligence algorithms for solving optimization problems: Applications and trends. *IEEE/CAA J. Autom. Sin.* **8**(10), 1627–1643. 10.1109/JAS.2021.1004129 (2021).

[9] Yildiz, B. S. A comparative investigation of eight recent population-based optimisation algorithms for mechanical and structural design problems. *Int. J. Veh. Des.* **73**(1), 208–218. 10.1504/IJVD.2017.082603 (2017).

[10] Govindaraj, V. & Ramasamy, J. V. Optimum detailed design of reinforced concrete continuous beams.pdf, vol 39, no. 4, 471–494 (2007).

[11] Yang, X. S., Bekdas, G. & Nigdeli, S. M. *Preface*, vol. 7. 2016. 10.1007/978-3-319-26245-1.

[12] Yang, X. S., Bekdas, G. & Nigdeli, S. M. Preface. *Modeling and Optimization in Science and Technologies* **7**, v–vi. 10.1007/978-3-319-26245-1 (2016).

[13] Kashani, A. R., Camp, C. V., Rostamian, M., Azizi, K. & Gandomi, A. H. Population-based optimization in structural engineering: A review. *Artif. Intell. Rev.* **55**(1), 345–452. 10.1007/s10462-021-10036-w (2022).

[14] Dirik, M. Comparison of recent meta-heuristic optimization algorithms using different benchmark functions. *J. Math. Sci. Modell.* **5**(3), 113–124. 10.33187/jmsm.1115792 (2022).

[15] Luo, Y., Liao, P., Pan, R., Zou, J. & Zhou, X. Effect of bar diameter on bond performance of helically ribbed GFRP bar to UHPC. *J. Build. Eng.* **91**, 109577. 10.1016/j.jobe.2024.109577 (2024).

[16] Zheng, H. et al. Mechanical properties and microstructure of waterborne polyurethane-modified cement composites as concrete repair mortar. *J. Build. Eng.* **84**, 108394. 10.1016/J.JOBE.2023.108394 (2024).

[17] Zheng, H. et al. Durability enhancement of cement-based repair mortars through waterborne polyurethane modification: Experimental characterization and molecular dynamics simulations. *Constr. Build. Mater.* **438**, 137204. 10.1016/J.CONBUILDMAT.2024.137204 (2024).

[18] Zheng, H. et al. Reaction molecular dynamics study of calcium alumino-silicate hydrate gel in the hydration deposition process at the calcium silicate hydrate interface: The influence of Al/Si. *J. Build. Eng.* **86**, 108823. 10.1016/j.jobe.2024.108823 (2024).

[19] Zheng, H. et al. Unveiling the dissolution mechanism of calcium ions from CSH substrates in Na_2_SO_4_ solution: Effects of Ca/Si ratio. *Appl. Surf. Sci.* **680**, 161443. 10.1016/j.apsusc.2024.161443 (2025).

[20] Alsattar, H. A., Zaidan, A. A. & Zaidan, B. B. Novel meta-heuristic bald eagle search optimisation algorithm. *Artif. Intell. Rev.* **53**(3), 2237–2264. 10.1007/s10462-019-09732-5 (2020).

[21] Zhang, Q., Gao, H., Zhan, Z. H., Li, J. & Zhang, H. Growth Optimizer: A powerful metaheuristic algorithm for solving continuous and discrete global optimization problems. *Knowl. Based Syst.* **261**, 110206. 10.1016/J.KNOSYS.2022.110206 (2023).

[22] Mirjalili, S. The ant lion optimizer. *Adv. Eng. Softw.* **83**, 80–98. 10.1016/j.advengsoft.2015.01.010 (2015).

[23] Xie, L. et al. Tuna swarm optimization: A novel swarm-based metaheuristic algorithm for global optimization. *Comput. Intell. Neurosci.*10.1155/2021/9210050 (2021). 34721567 10.1155/2021/9210050 PMC8550856

[24] Kaur, S., Awasthi, L. K., Sangal, A. L. & Dhiman, G. Tunicate swarm algorithm: A new bio-inspired based metaheuristic paradigm for global optimization. *Eng. Appl. Artif. Intell.* **90**(February), 103541. 10.1016/j.engappai.2020.103541 (2020).

[25] Heidari, A. A. et al. Harris hawks optimization: Algorithm and applications. *Future Gener. Comput. Syst.* **97**, 849–872. 10.1016/j.future.2019.02.028 (2019).

[26] Abdollahzadeh, B. Artificial gorilla troops optimizer: A new nature‐inspired metaheuristic algorithm for global optimization problems. *Int. J. Intell. Syst.* **36**(April), 5887–5958. 10.1002/int.22535 (2021).

[27] H. Peraza-Vázquez, A. F. Peña-Delgado, G. Echavarría-Castillo, A. B. Morales-Cepeda, J. Velasco-Álvarez, and F. Ruiz-Perez, “A Bio-Inspired Method for Engineering Design Optimization Inspired by Dingoes Hunting Strategies,” *Math Probl Eng*, vol. 2021, 2021, 10.1155/2021/9107547.

[28] Kennedy, J. & Eberhart, R. Particle swarm optimization. In *Proceedings of ICNN’95 - International Conference on Neural Networks*, Vol 4, 1942–1948. 10.1109/ICNN.1995.488968 (1995).

[29] Mirjalili, S., Mirjalili, S. M. & Lewis, A. Grey wolf optimizer. *Adv. Eng. Softw.* **69**, 46–61. 10.1016/j.advengsoft.2013.12.007 (2014).

[30] Kaveh, A. & Mahdavi, V. R. Colliding bodies optimization: A novel meta-heuristic method. *Comput. Struct.* **139**, 18–27. 10.1016/j.compstruc.2014.04.005 (2014).

[31] Cuevas, E. & Cienfuegos, M. A new algorithm inspired in the behavior of the social-spider for constrained optimization. *Expert Syst. Appl.* **41**(2), 412–425. 10.1016/j.eswa.2013.07.067 (2014).

[32] Cuong-Le, T. et al. A novel version of Cuckoo search algorithm for solving optimization problems. *Expert Syst. Appl.* **186**, 115669. 10.1016/j.eswa.2021.115669 (2021).

[33] Mohapatra, S. & Mohapatra, P. American zebra optimization algorithm for global optimization problems. *Sci. Rep.*10.1038/s41598-023-31876-2 (2023). 36997597 10.1038/s41598-023-31876-2 PMC10063666

[34] Akay, B. & Karaboga, D. Artificial bee colony algorithm for large-scale problems and engineering design optimization. *J. Intell. Manuf.* **23**(4), 1001–1014. 10.1007/s10845-010-0393-4 (2012).

[35] Pathak, V. K. & Srivastava, A. K. A novel upgraded bat algorithm based on cuckoo search and Sugeno inertia weight for large scale and constrained engineering design optimization problems. *Eng. Comput.* **38**(2), 1731–1758. 10.1007/s00366-020-01127-3 (2022).

[36] Khalilpourazari, S. & Khalilpourazary, S. An efficient hybrid algorithm based on water cycle and moth-flame optimization algorithms for solving numerical and constrained engineering optimization problems. *Soft Comput.* **23**(5), 1699–1722. 10.1007/s00500-017-2894-y (2019).

[37] Sun, P., Liu, H., Zhang, Y., Tu, L. & Meng, Q. An intensify atom search optimization for engineering design problems. *Appl. Math. Model.* **89**, 837–859. 10.1016/j.apm.2020.07.052 (2021).

[38] Gupta, S. et al. Comparison of metaheuristic optimization algorithms for solving constrained mechanical design optimization problems. *Expert Syst. Appl.* **183**(June), 115351. 10.1016/j.eswa.2021.115351 (2021).

[39] Dalirinia, E., Jalali, M., Yaghoobi, M. & Tabatabaee, H. Lotus effect optimization algorithm (LEA): A lotus nature-inspired algorithm for engineering design optimization. *J. Supercomput.*10.1007/s11227-023-05513-8 (2023).

[40] Karami, H., Anaraki, M. V., Farzin, S. & Mirjalili, S. Flow direction algorithm (FDA): A novel optimization approach for solving optimization problems. *Comput. Ind. Eng.* **156**(March), 107224. 10.1016/j.cie.2021.107224 (2021).

[41] Hashim, F. A., Mostafa, R. R., Khurma, R. A., Qaddoura, R. & Castillo, P. A. A new approach for solving global optimization and engineering problems based on modified sea horse optimizer. *J. Comput. Des. Eng.* **11**(1), 73–98. 10.1093/jcde/qwae001 (2024).

[42] Ong, P., Ho, C. S., Daniel, D. & Sheng, V. CCAM communications in computational and applied an improved cuckoo search algorithm for design optimization of structural engineering problems. **2**(1), (2020).

[43] Digehsara, P. A., Chegini, S. N., Bagheri, A. & Roknsaraei, M. P. An improved particle swarm optimization based on the reinforcement of the population initialization phase by scrambled Halton sequence. *Cogent Eng.*10.1080/23311916.2020.1737383 (2020).

[44] Shang, C., ting Zhou, T. & Liu, S. Optimization of complex engineering problems using modified sine cosine algorithm. *Sci. Rep.* **12**(1), 1–25. 10.1038/s41598-022-24840-z (2022). 36443452 10.1038/s41598-022-24840-z PMC9705278

[45] Yu, H., Zhao, N., Wang, P., Chen, H. & Li, C. Chaos-enhanced synchronized bat optimizer. *Appl. Math. Model.* **77**, 1201–1215. 10.1016/j.apm.2019.09.029 (2020).

[46] Abualigah, L. et al. Aquila optimizer: A novel meta-heuristic optimization algorithm. *Comput. Ind. Eng* **157**(October), 107250. 10.1016/j.cie.2021.107250 (2021).

[47] Abualigah, L., Diabat, A., Mirjalili, S., Abd Elaziz, M. & Gandomi, A. H. The arithmetic optimization algorithm. *Comput Methods Appl Mech Eng* **376**, 113609. 10.1016/j.cma.2020.113609 (2021).

[48] Fauzi, H. & Batool, U. A three-bar truss design using single-solution simulated Kalman filter optimizer. *Mekatronika* **1**(2), 98–102. 10.15282/mekatronika.v1i2.4991 (2019).

[49] Ahmadianfar, I., Heidari, A. A., Noshadian, S., Chen, H. & Gandomi, A. H. INFO: An efficient optimization algorithm based on weighted mean of vectors. *Expert Syst. Appl.* **195**(June), 116516. 10.1016/j.eswa.2022.116516 (2022).

[50] Ahmadianfar, I., Heidari, A. A., Gandomi, A. H., Chu, X. & Chen, H. RUN beyond the metaphor: An efficient optimization algorithm based on Runge Kutta method. *Expert Syst. Appl.* **181**(April), 115079. 10.1016/j.eswa.2021.115079 (2021).

[51] Bayzidi, H., Talatahari, S., Saraee, M. & Lamarche, C. P. Social network search for solving engineering optimization problems. *Comput. Intell. Neurosci.*10.1155/2021/8548639 (2021). 34630556 10.1155/2021/8548639 PMC8497131

[52] Yildiz, B. S., Pholdee, N., Bureerat, S., Yildiz, A. R. & Sait, S. M. Enhanced grasshopper optimization algorithm using elite opposition-based learning for solving real-world engineering problems. *Eng. Comput.* **38**(5), 4207–4219. 10.1007/s00366-021-01368-w (2022).

[53] Altay, E. V., Altay, O. & Özçevik, Y. A comparative study of metaheuristic optimization algorithms for solving real-world engineering design problems. *Comput. Model. Eng. Sci.* **139**(1), 1039–1094. 10.32604/cmes.2023.029404 (2024).

[54] Siddavaatam, P. & Sedaghat, R. *A New Bio-heuristic Hybrid Optimization for Constrained Continuous Problems*, Vol. 12620 (LNCS, 2021). 10.1007/978-3-662-63170-6_5.

[55] Yuan, Y., Ren, J., Zu, J. & Mu, X. An adaptive instinctive reaction strategy based on Harris hawks optimization algorithm for numerical optimization problems. *AIP Adv.*10.1063/5.0035635 (2021).

[56] Nama, S. & Saha, A. K. A bio-inspired multi-population-based adaptive backtracking search algorithm. *Cognit. Comput.* **14**(2), 900–925. 10.1007/s12559-021-09984-w (2022). 35126764 10.1007/s12559-021-09984-w PMC8800854

[57] Nama, S., Saha, A. K. & Sharma, S. A novel improved symbiotic organisms search algorithm. *Comput. Intell.* **38**(3), 947–977. 10.1111/coin.12290 (2022). 10.1007/s12652-021-03183-z PMC8036246 33868507

[58] Coello Coello, C. A. Use of a self-adaptive penalty approach for engineering optimization problems. *Comput. Ind.* **41**(2), 113–127. 10.1016/S0166-3615(99)00046-9 (2000).

[59] Fleury, C. & Braibant, V. Structural optimization: A new dual method using mixed variables. *Int. J. Numer. Methods Eng.* **23**(3), 409–428. 10.1002/nme.1620230307 (1986).

[60] Gold, S., Krishnamurty, S. Trade-offs in robust engineering design. In *ASME 1997 Design Engineering Technical Conferences* (1997).

[61] *2020 IEEE Congress on Evolutionary Computation (CEC): 2020 conference proceedings*. (IEEE, 2020).

[62] Derrac, J., García, S., Molina, D. & Herrera, F. A practical tutorial on the use of nonparametric statistical tests as a methodology for comparing evolutionary and swarm intelligence algorithms. *Swarm Evol. Comput.* **1**(1), 3–18. 10.1016/j.swevo.2011.02.002 (2011).

[63] García, S., Fernández, A., Luengo, J. & Herrera, F. Advanced nonparametric tests for multiple comparisons in the design of experiments in computational intelligence and data mining: Experimental analysis of power. *Inf. Sci. (N. Y.)* **180**(10), 2044–2064. 10.1016/j.ins.2009.12.010 (2010).

